# Influence of Ventilation Rate and Indoor Air Mixing on Ozone–Human Skin Chemistry

**DOI:** 10.1021/acsestair.5c00433

**Published:** 2026-03-30

**Authors:** Tatjana Arnoldi-Meadows, Nijing Wang, Gabriel Bekö, Marouane Merizak, Pawel Wargocki, Meixia Zhang, Shen Yang, Dusan Licina, Jonathan Williams

**Affiliations:** † 28309 Max Planck Institute for Chemistry, Hahn-Meitner Weg 1, 55128 Mainz, Germany; ‡ International Centre for Indoor Environment and Energy, Department of Environmental and Resource Engineering, 5205 Technical University of Denmark, 2800 Kongens Lyngby, Denmark; § Human-Oriented Built Environment Lab, School of Architecture, 27218 Civil and Environmental Engineering, École Polytechnique Fédérale de Lausanne (EPFL), 1015 Lausanne, Switzerland; ∥ School of Mechanical Engineering, 47833 Beijing Institute of Technology, Beijing 100081, China; ⊥ School of Architecture, Southeast University, 210096 Nanjing, China; # Climate and Atmosphere Research Center, The Cyprus Institute, 1645 Nicosia, Cyprus

**Keywords:** indoor air chemistry, air mixing, ventilation, ozone, VOCs, skin emissions, 4-OPA, 6-MHO

## Abstract

Human exposure to indoor air pollutants can be impacted both by the rate of outdoor air ventilation and by indoor air mixing driven by fans. To elucidate these effects, we monitored human chemical emissions in an occupied climate chamber, under three air change rates (ACRs: 0.5, 2.5, and 3 h^–1^), and, for the highest ACR, with and without mixing fans. Experiments were conducted with ozone present at all ACRs (0.5, 1.5, and 3 h^–1^), with additional no-ozone conditions at the two higher ACRs. Volatile organic compounds (VOC) were measured with chemical ionization mass spectrometry (CIMS) using two different reagents (H_3_O^+^ and NO^+^). Increasing the ACR reduced the steady-state mixing ratios of all measured compounds. When mixing fans were switched off, ozone volume mixing ratios (VMRs) increased from 25 to 35 ppb, while 6-MHO levels decreased ∼20% from 1.4 to 1.1 ppb. Changes in 6-MHO (6-methyl-5-hepten-2-one) and O_3_ resulted in a relatively unchanged 4-OPA (4-oxopentanal) production rate, resulting in a VMR of 1.8 ppb. Future modeling of this mechanism is needed. These results emphasize the importance of both ventilation (ACR) and air mixing (fans) in determining indoor chemical concentrations.

## Graphical abstract

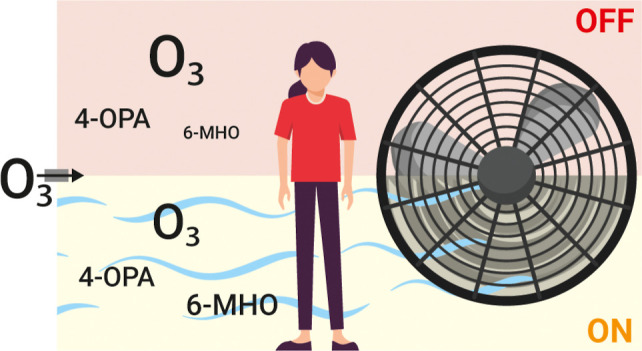

## Introduction

Surveys of human activity patterns in the US, Canada, and Germany indicate that people spend a large proportion (>85%) of their daily life within indoor or enclosed environments.^1–3^ Therefore, the composition of indoor air critically determines the chemical exposure of any individual throughout their lifetime. Some outdoor pollutants, such as PM_2.5_ and ozone, enter buildings through infiltration and active ventilation.^4^ Once inside, ozone can initiate chemical reactions, in particular by reacting with compounds on surfaces and in the gas phase with compounds that have double bonds. These reactions form radicals, oxygenated products, and particles.^5–9^ Because of these reactions, typical indoor ozone concentrations are on average 25% of the outside concentration.^10^

In contrast many chemicals are emitted indoors and can lead to higher concentrations than occurring outdoors.^11^ Important continuous sources of indoor pollutants include emission from building materials, furnishings, and paints^12–15^ but intermittent strong short-term sources from cleaning or cooking are known to generate extreme indoor levels of a multitude of compounds with a variety of toxicities.^16,17^ Also, the usage of personal care products releases high amounts of VOCs.^18^ Among all emission sources, humans themselves are ubiquitous and continuous emitters of volatile and semivolatile organic compounds. Recent research has comprehensively characterized the human emissions from breath and the skin,^5–8,17,19–23^ revealing that ozone profoundly affects the human volatilome composition. In particular, reaction of ozone with reactive compounds contained in human skin oil, such as squalene, generates a host of volatile products including 6-methyl-5-hepten-2-one (6-MHO), leading to an increase in the overall reactivity of chemicals in the immediate vicinity of people.^5,22^ Some of these products can react further with ozone (via Criegee reactions) in the gas phase yielding yet more oxygenated products including 4-oxopentanal (4-OPA) and even hydroxyl radicals (OH).^22,24^ OH radicals enhance oxidation, further generating yet more products from indoor pollutants. Similarly, reactive organic compounds in human breath such as isoprene can also react with ozone and OH radicals to produce additional complex oxygenated products. The combination of these effects has been shown in models to generate a complex cocktail of chemical compounds and strong concentration gradients for radicals and reactants around the person.^25^ Some of these product species may have implications for human health upon exposure through breathing or the skin. Presently, the acute and chronic human health impacts of most indoor oxidation products are poorly understood. However, exposure to one specific oxidation product, 4-OPA, has been shown to lead to sensory irritation and allergic responses in mice.^26^ It should be noted that the concentrations used in mice studies exceed commonly measured mixing ratios. Additionally, elevated aldehyde (also produced through oxidation of skin oils^27,28^ ) and other volatile organic compounds (VOC) levels were found to correlate with the risk of the sick building syndrome.^29^ Only an overall trend linked to an increase of SBS symptoms could be found, but no quantitative estimates could be made due to a lack of exposure response functions. This underlines the need for more studies to evaluate the potential risks of VOCs in indoor environments. Due to the relative ease of its measurement, carbon dioxide, CO_2_, is often used as a proxy for the complex indoor air quality in occupied rooms. Many European countries use national guidelines, hygienic guide values, or ventilation standards that reference CO_2_ (often around 1000–1500 ppm) as comfort or IAQ indicators. However, it should be noted that the CO_2_ concentrations indicate only levels of occupancy and do not reflect the complexity of the VOC mixtures, the specific health effects of the mixture, or the physicochemical behavior of the VOC in the particular indoor space.

Indoor pollutant concentrations in a person’s breathing zone depend on both ventilation (air change rate, ACR) and air mixing (turbulence induced by fans or occupant activity). Higher ACRs generally dilute indoor pollutants if the incoming air is clean but can also transport additional ozone that fuels secondary chemistry. On the other hand, airflow patterns affect surface interactions and gradients near occupants. At low air mixing, thermal plumes from body heat dominate air movement, generating localized flows from the lower to upper body before dispersing (simulated in Zannoni et al.^22^). In contrast, mixing fans are often used in chamber studies to homogenize the air within,^19^ but this may alter surface chemistry and pollutant formation rates.^9^ In summary, the chemical composition of air surrounding occupants of indoor spaces is determined by both the air change rate, which controls the exchange of indoor and outdoor air, and air mixing, which influences how pollutants and reactive species are distributed within the space. Yet, the combined influence of ventilation rate and air mixing on human-derived indoor chemistry has not been systematically examined and quantified. A previous study showed the influence of ACR on VOCs and ozone loss on worn shirts.^30^ Ozone removal was dominated by ACR and surface loss, and only 4% of ozone was lost in the gas phase. Slightly higher ozone/t-shirt deposition was reported compared to earlier literature, and it was suggested that this might be due to the presence of mixing fans.

In this study, we examine how varying ventilation rates (at ACRs of 0.5 h^–1^, 1.5 h^–1^, and 3.0 h^–1^) and fan operation affect ozone, 6-MHO, and 4-OPA levels in a climate chamber occupied by six people. By comparing these conditions, we reveal how ventilation and indoor airflow shape the formation, transformation, and removal of reactive organic compounds in occupied indoor environments. Additionally, two different online chemical mass spectrometers, which are now widely used to track the concentrations of volatile organic compounds in real time, are compared. Most systems use H_3_O^+^ ions to transfer a proton to the neutral molecule, while in some cases NO^+^ is used to differentiate species that can otherwise not be distinguished by the H_3_O^+^ method. Rarely are these techniques used in parallel to allow for comparison.

## Materials and Methods

### Experimental Setup

Measurements were performed in a climate-controlled chamber of the Human-Oriented Built Environment Lab (HOBEL) in Fribourg, Switzerland. The chamber had a volume of 60 m^3^ with a floor area of 25 m^2^. Further information on the chamber, including an in-depth description of the experimental setup and running conditions is given elsewhere.^9^ Briefly, the air change rates were as follows: 0.55 h^–1^ and 0.55 h^–1^ (referred to as 0.5 h^–1^), 1.47 h^–1^ and 1.49 h^–1^ (referred to as 1.5 h^–1^), and 2.98 h^–1^ and 2.98 h^–1^ (referred to as 3 h^–1^), for each experiment and its replicate. Additional runs with fans off featured calculated ACRs of 2.9 h^–1^ and 2.94 h^–1^ for the replicate (referred to as 3 h^–1^ fans off). The reported and measured values are the same as reported in Yang et al. 2024.^9^

The CO_2_ mixing ratio was measured by a Picarro Cavity Ring Down Spectrometer (Picarro, Inc., Santa Clara, USA) using a 1.27 cm diameter (1/2 in.) Teflon inlet line of 3.85 m in total length. The same inlet line was used by the two online VOC instruments, each sampling from the combined flow, so that wall losses were minimized and measurements were collocated. This flow is generated by a separate sampling pump (see Figure 1) and provided an overall flow through the main inlet line of 12.5 L/min. Several other parameters such as temperature, humidity, and ozone levels were monitored (detailed elsewhere^9^). The ozone input mixing ratio (generated by a Jelight 600 UV ozone generator) was adjusted to keep the resulting chamber ozone levels constant at ∼25 ppb for the experiments focusing on the effect of different ACRs. For the experiment focusing on the effect of fans, the ozone generation rate was kept as it was for 3 air change rates per hour with fans on, resulting in elevated ozone levels of ∼35 ppb. Ozone was measured at both the injection line in the supply air diffuser mounted on the ceiling and the chamber’s air exhaust located on a table near a wall with a distance to the supply air diffuser of ∼2 m; their volume mixing ratio (VMR) difference at steady state represents the ozone loss inside the chamber.

**Figure 1:**
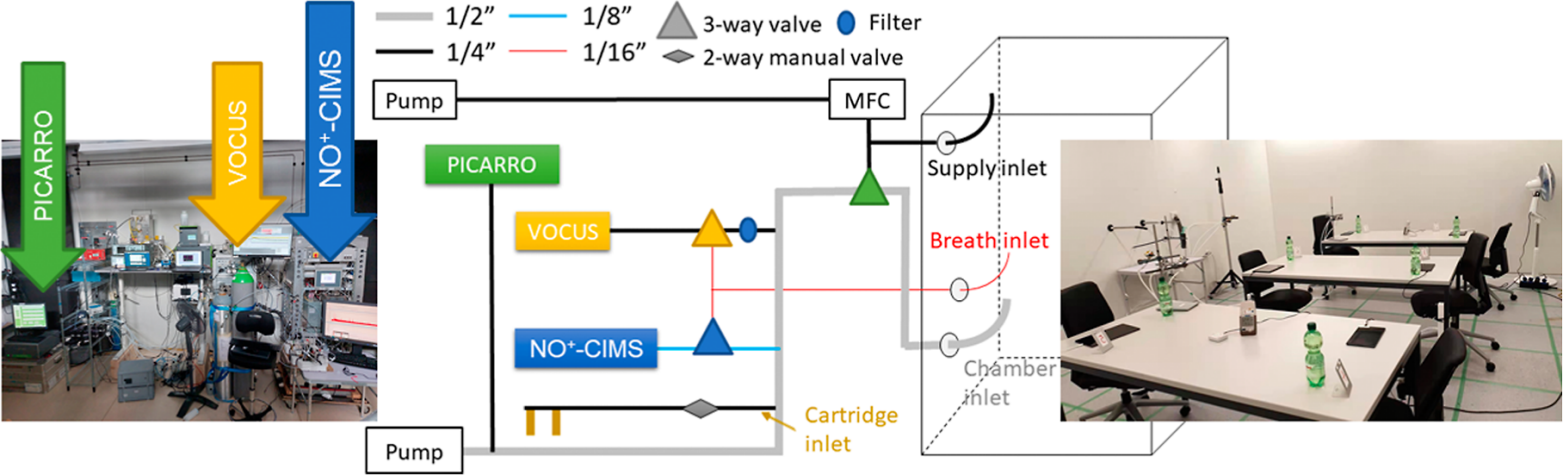
Instrumentation site, flow scheme, and chamber used during the campaign. The picture on the left side shows the instrumental room (outside chamber) with the 2 mass spectrometers and the Picarro (and other instruments not used in this study). The picture on the right shows the unoccupied climate chamber. In the middle the inlet flow scheme is shown. Gray lines are 1/2″ lines, black lines are 1/4″ lines, 1/8″ lines are shown in blue, and 1/16″ lines are shown in red. The filled blue oval shows the PTFE filter; 3-way-valves are shown as triangles; the 2-way manual valve is shown as a gray diamond. The mass flow controller, controlling the flow through the supply inlet line is shown as MFC.

The chamber contained only four tables and six chairs to minimize nonhuman emissions or surface sinks. The inlet lines of the various instruments were placed opposite the chamber entrance. The supply air entering the chamber was outside air, filtered with three-stage filtration (described elsewhere^9^) to remove any potential particle and VOC contamination; however, outside ozone was not removed by the filtering system. Nonetheless, the supply air was also periodically measured to account for any possible contamination via a three-way Teflon valve (Galtek Solenoid Valves, Entegris, Inc.). For experiments involving ventilation rates, 2 fans were placed in opposite corners of the room facing the wall in order to better distribute the air in the chamber (see Figure 1; the other fan not shown on the opposite side of the room). When switched on, the fans were run in the highest speed mode at 1350 m^3^

### Instrumentation

A Vocus (Tofwerk AG and Aerodyne Research, Inc.) PTR-ToF-MS and a NO^+^-CIMS (8000, Ionicon Analytik GmbH, Innsbruck, Austria) were used to measure VOCs in the chamber in real time. A Picarro G 2401 was used to measure the CO_2_ concentration and cartridges were taken (not used in this study but influenced CO_2_ concentration when opening the valve). All of the lines shown in Figure 1 were Teflon lines. The 1/16″ line in red allowed breath samples to be taken periodically at the end of each experiment (not part of this study). We performed measurements, either in the supply air, or chamber air using a 3-way valve (green). To ensure no dead volume was present in the supply inlet line, a pump, and a mass flow controller (MFC) were installed. Similarly, to avoid a dead volume and to increase the flow through the chamber inlet (in order to reduce wall losses in the inlet), an additional pump was installed; see Figure 1. The residence time of VOCs in the inlet (from chamber to instruments) was 6 s (checked with acetone). The length of the air supply inlet to the half inch main sampling inlet (green 3-way valve) was 165 cm (all lengths rounded to 5 cm). The length of the chamber inlet to the green 3-way valve was 180 cm. The distance from this green 3-way valve to the Vocus’ T-piece was 55 cm. The distance from this T-piece (including a PTFE filter to prevent particles to enter the Vocus) to the yellow 3-way valve for breath sampling was 65 cm. The length of the half inch line from the green 3-way valve to the NO^+^-CIMS’ T-piece was 90 cm. The 1/8″ inlet from the half inch line to the blue 3-way valve was 70 cm.

The Vocus was operated as a proton-transfer reaction time-of-flight mass spectrometer (PTR-ToF-MS) in the H_3_O^+^ mode. VOCs with a proton affinity higher than that of water (691 kJ/mol) undergo a proton-transfer reaction in which the hydronium ions lose an H^+^ ion and ionize VOCs to form (VOC)­H^+^ ions. The Vocus features a focusing ion–molecule reactor (FIMR), which increases the detection sensitivity compared to a typical PTR-MS. Since using hydronium ions for VOC ionization only provides specific sum molecular formulas without functional group details; additional measurements were used to give more chemical information. By using another Chemical Ionization Mass Spectrometer (CIMS) in the NO^+^ mode, aldehydes and ketones could be resolved. Additionally, isoprene, which is known to be influenced by aldehyde fragments when using hydronium ions for ionization in indoor environments,^31,32^ can be measured more specifically when using NO^+^ for ionization. The NO^+^-ToF-MS instrument is a CIMS, similar in principle to the aforementioned Vocus, but with NO^+^ as the reagent ion. The ionization reactions of NO^+^ are slightly more complicated as they can proceed through hydride abstraction, clustering, and charge transfer. For example, NO^+^ ionizes aldehydes mainly via hydride abstraction, whereas ketones and NO^+^ tend to form a cluster leading to different product ions.^33,34^

The operating settings for the two instruments were the following: For the NO^+^-CIMS, the experiments were conducted with the drift tube set to 400 V so the E/N (electric field to gas number density) was 92 Td, lower in comparison to the Vocus. To implement the NO^+^ chemical ionization, synthetic air instead of water vapor was introduced into the ion source, and the source parameters were tuned to achieve a low contribution of impurity ions and high counts of NO^+^. The time resolution was set to one mass spectrum every 12 s. The mass resolution was ∼3200 at mass 100 amu. Data analysis of NO^+^-CIMS was performed using IDA (Ionicon Data Analyzer, version 2.1.1.4; Ionicon Analytik, Austria). The settings for the Vocus instrument were as follows: a reactor temperature of 60 °C, an ion molecule reaction region—region of proton-transfer reaction pressure of 2 mbar, and a potential of 475 V along the IMR. Further information on the settings can be found in detail in the Supporting Information. This results in an E/N of around 110 Td. The time resolution in this study was set to one mass spectrum every 4 s, within a mass range of 11 to 500 *m*/*z*. The achieved mass resolution was around 10,000 at 500 *m*/*z*. The analysis for Vocus data was conducted with Tofware version 3.2.5. The peaks were selected manually. The Vocus creates a cut off (in order to protect the detector from too many ions) in our case at mass 50 amu. The resulting transmission curve is shown in Supporting Information. Additionally, the Vocus has some issues with fragmentation, especially for the lighter ions which might also be influenced by fragmentation of heavier ions. It should be noted that the theoretically determined mixing ratios of not calibrated compounds can only be seen as a lower limit, due to the fact that the produced fragments were not included in the parent mass signal.

Comprehensive calibrations for both CIMS instruments were performed twice during the experiment using a gas mixture containing 18 different VOCs (Apel-Riemer Environmental Inc.; USA). After the experimental campaign, two further calibrations, one with 6-MHO and one with 4-OPA, were conducted. Details of the limit of detection, measurement uncertainties, calibration slopes, and transmission curves are summarized in the Supporting Information.

### Experimental Procedure

The participants were given specially selected odorless personal care products (such as shampoo, shower gel, and soap). They were asked to take a shower the day before each experimental day and were asked not to use any personal care products other than those provided. They were also provided with standardized clothing (T-shirts and trousers). The clothes were prewashed with fragrance-free detergent. Additionally, participants were instructed not to eat spicy food or smelly food items such as garlic. For each experiment, a replicate was performed on another day with the same participants.

For the low ACR experiments (0.5 h^–1^), the participants stayed in the chamber for 5 consecutive hours with a short restroom break (less than 10 min) after 2.5 h. This was necessary due to the long time needed to reach steady-state conditions at low ACR. For all other experiments, the participants stayed inside the chamber in the morning for 3 h, left the chamber for lunch (circa 1 h), and reentered the chamber to stay for another 3 h. In these experiments, the participants were given the same type of lunch (sandwich) every day in order to allow intercomparison of the data after lunch. The experiments were separated into two sessions, no-ozone conditions in the morning (mimicking low outdoor ozone levels) and added ozone conditions in the afternoon (mimicking high outdoor ozone levels), except for the low ACR experiments, where ozone was injected from the morning. The injection of ozone was always done 10 min after participants entered the chamber. The ozone input was adjusted to obtain the same ozone levels of 25 ppb in the chamber for different ACRs (inlet ozone mixing ratio for ACRs of 0.5 h^–1^: 230 ppb; 1.5 h^–1^: 100 ppb; 3 h^–1^: 65 ppb) and were kept at that setting when testing the effect of fans (inlet ozone mixing ratio: 65 ppb for an ACR of 3 h^–1^), which led to an increased ozone level of 35 ppb.

## Results and Discussion

### Intercomparison between Two CIMS Instruments

As in this study, two different CIMS instruments were used to measure VOCs (Vocus and NO^+^-CIMS), each with distinct advantages and limitations, an intercomparison of the measured volume mixing ratios (VMRs) was conducted to assess data consistency. Table 1 compares the mean VMRs of selected compounds measured with the two instruments during the afternoon period (when ozone was present; specific times used to calculate mean VMRs are given in the Supporting Information in Table S5). For acetone and dimethyl sulfide (DMS), which were calibrated using the same standard and synchronized calibration procedures, the agreement between the two instruments was excellent, with differences typically <10% in the linear calibration range. This indicates good overall consistency between Vocus and NO^+^-CIMS for these well-characterized compounds. The calibrations for the VOCs shown in Table S1 in the Supporting Information range between 2.5 and 10 ppb. For 6-MHO and 4-OPA, the calibration range was between 1 and 20 ppb (calibrations for 6-MHO and 4-OPA were performed after the calibration for each instrument independently). For 6-MHO and 4-OPA, the Vocus reported values of 70–80% and 65–75%, respectively, of those measured by the NO^+^-CIMS. This deviation is most likely due to additional compounds or fragments landing on the same masses of 6-MHO and 4-OPA for NO^+^-CIMS. In addition, differences in the calibration procedure which was performed for the two instruments at different times and locations might also play a role.

**Table 1:** Comparison of Average Mixing Ratios Taken from the Last 10 min of Each Experiment (Specific Times in SI Table 5) for the Two Different Measurement Methods^b^

compound chemical formular Vocus/NO^+^-CIMS	ACR	Vocus benchmark, replicate VMR in ppb	NO^+^-CIMS benchmark, replicate VMR in ppb
**6-MHO**	ACR = 0.5 h^–1^	**4.19, 4.30**	5.47, 4.91
C_8_H_15_O^+^/C_8_H_14_O^+^	ACR = 1.5 h^–1^	**3.05, 3.10**	4.41, 4.12
	ACR = 3 h^–1^	**1.86, 2.13**	2.29, 2.65
**4-OPA**	ACR = 0.5 h^–1^	**8.00, 7.72**	11.31, 10.62
C_5_H_9_O_2_^+^/C_5_H_8_O_2_^+^	ACR = 1.5 h^–1^	**3.75, 4.09**	5.22, 5.68
	ACR = 3 h^–1^	**2.26, 2.60**	3.30, 3.93
**GA**	ACR = 0.5 h^–1^	**0.08, 0.08**	none
C_13_H_23_O^+^/–	ACR = 1.5 h^–1^	**0.08, 0.08**	
	ACR = 3 h^–1^	**0.05, 0.06**	
**acetone**	ACR = 0.5 h^–1^	55.69, 68.34	51.87, 60.22
C_3_H_7_O^+^/(C_3_H_6_O)NO^+^	ACR = 1.5 h^–1^	28.17, 34.64	24.70, 30.48
	ACR = 3 h^–1^	15.64, 20.21	13.90, 18.44
**DMS**	ACR = 0.5 h^–1^	0.14, 0.20	0.15, 0.20
C_2_H_7_S^+^/C_2_H_6_S^+^	ACR = 1.5 h^–1^	0.08, 0.08	0.08, 0.06
	ACR = 3 h^–1^	0.04, 0.04	0.03, 0.03
**isoprene**	ACR = 0.5 h^–1^	23.49, 23.42	**6.15, 6.07**
C_5_H_9_^+^/C_5_H_8_^+^	ACR = 1.5 h^–1^	11.42, 11.02	**2.53, 2.83**
	ACR = 3 h^–1^	7.02, 7.63	**1.53, 1.77**
**Aldehydes**			
**hexanal**	ACR = 0.5 h^–1^	2.17 (0.49), 2.49 (0.57)	**1.82, 1.82**
C_6_H_13_O^+^/C_6_H_11_O^+^	ACR = 1.5 h^–1^	1.01 (0.23), 0.98 (0.22)	**0.92, 0.83**
	ACR = 3 h^–1^	0.48 (0.11), 0.51 (0.12)	**0.50, 0.51**
**heptanal**	ACR = 0.5 h^–1^	0.88 (0.27), 1.06 (0.32)	**0.89, 0.89**
C_7_H_15_O^+^/C_7_H_13_O^+^	ACR = 1.5 h^–1^	0.42 (0.13), 0.43 (0.13)	**0.44, 0.42**
	ACR = 3 h^–1^	0.21 (0.06), 0.23 (0.07)	**0.25, 0.27**
**octanal**	ACR = 0.5 h^–1^	**0.62 (0.32), 0.70 (0.36)**	0.54, 0.60
C_8_H_17_O^+^/C_8_H_15_O^+^	ACR = 1.5 h^–1^	**0.32 (0.16), 0.35 (0.18)**	0.17, 0.17
	ACR = 3 h^–1^	**0.19 (0.09), 0.21 (0.11)**	0.14, 0.14
**nonanal**	ACR = 0.5 h^–1^	1.81 (1.50), 1.83 (1.52)	**3.48, 3.14**
C_9_H_19_O^+^/C_9_H_17_O^+^	ACR = 1.5 h^–1^	0.99 (0.82), 0.93 (0.77)	**1.95, 1.59**
	ACR = 3 h^–1^	0.60 (0.50), 0.65 (0.54)	**1.27, 1.31**
**decanal**	ACR = 0.5 h^–1^	(1.08), (1.16)^a^	**2.10, 1.99**
C_10_H_21_O^+^/C_10_H_19_O^+^	ACR = 1.5 h^–1^	(0.62), (0.72)^a^	**1.27, 1.23**
	ACR = 3 h^–1^	(0.42), (0.50)^a^	**0.89, 0.97**

a Only the parent ion considered, as the fragmentation test was not successful due to potential decanal solution contamination.

b Highlighted in bold: data used for analysis and figures. Aldehyde data from Vocus adapted to the observed fragmentation ratio from the main fragment (aldehyde losing a water molecule) from the aldehyde fragmentation test shown in the Supporting Information. Data in brackets original data without considering fragment ratio.

However, for compounds with lower VMRs, the NO^+^-CIMS consistently reported lower values than that of the Vocus. This discrepancy may arise from operation outside the linear response range of the calibration or from interfering fragmentation pathways in the Vocus instrument. One compound that was not measured by the NO^+^-CIMS most likely due to the concentration being too low and not exceeding the limit of detection was geranyl acetone (GA); thus, it was only measured by the Vocus. However, the data only showed a weak dependency for a change in ACR. Thus, we instead focused on 6-MHO and 4-OPA in this study, which behaved consistently and were calibrated.

It should be noted that the Vocus instrument is prone to the fragmentation of aldehydes. Parent ion fragment into lower-mass fragments, especially with the used big segmented quadrupole (BSQ, a part of the instrument that is necessary to reduce the signal of small ions in order to protect the detector) settings.^32^ This is likely due to a high potential difference at the entrance of the BSQ (voltages shown in the Supporting Information) that seems to trigger fragmentation, which is discussed in Coggon et al.^32^ Thus, the measured VMR of “isoprene” showed a large difference between the two instruments, as expected: the Vocus detects aldehyde fragments which overlap with the exact mass of isoprene’s molecular ion, leading to a significant interference and overestimation. In contrast, for the NO^+^-CIMS aldehyde fragments do not disturb the measurement of isoprene (measured at *m*/*z* = 68.06).

For better quantifying the aldehyde signals, multiplication factors derived from aldehyde fragmentation tests (further information in the Supporting Information) were used for calculating the mixing ratios of aldehydes measured by Vocus. The approach used still results in a systematic underestimation in the Vocus data for highly fragmenting aldehydes, as smaller fragments were not included. While aldehyde fragmentation was significantly lower in NO + -CIMS compared to Vocus, fragment signals were excluded from quantification due to a low correlation with parent ions, leading to reported values that should be considered lower limits. With this approach, the overall agreement of the hexanal and heptanal is generally good (difference mostly <20%); for octanal, nonanal, and decanal the discrepancy exceeded ±50%. For octanal, this can be explained by (a) a higher limit of detection of the NO^+^- CIMS compared to the Vocus and (b) an influence of other compounds such as ketones or alcohols lying on the same mass as the parental mass of octanal for Vocus. For nonanal, this discrepancy can be explained by a high fragmentation fraction on *m*/*z* 69, which was not included in the multiplication factor for Vocus data. The discrepancy of decanal can be explained by not considering any multiplication factor to consider the fragments for Vocus data, as the fragmentation test failed for this compound. The VOCs and the results from the instrument considered for all plots are highlighted in bold in Table 1.

### Dynamics of Indoor VOCs Emitted via Breath

Figure 2 shows CO_2_ and isoprene which are typical breath compounds from humans for three experiments (a) ACR = 1.5 h^–1^ with fans on, (b) ACR = 3 h^–1^ with fans on, and (c) ACR = 3 h^–1^ with fans off. When the participants entered the chamber, both CO_2_ and isoprene exhibited a clear increase in the VMRs, reflecting their primary emission from humans. A sharp rise in VMRs was observed when participants were instructed to move, consistent with the increased metabolic rate and isoprene being released from moving muscles.^35^ Increasing the air change rate (ACR) from 1.5 h^–1^ (Figure 2a) to 3 h^–1^ (Figure 2b) resulted in a significant reduction in the VMRs of both compounds, confirming the expected dilution effect of enhanced ventilation. When comparing conditions with identical ACR (3 h^–1^) but differing fan operation—fans on (Figure 2b) versus fans off (Figure 2c)—the absence of fans led to greater temporal variability in VMR measurements, indicating less homogeneous air mixing and the presence of localized concentration gradients, despite equivalent overall ventilation rates (ACRs). The disturbances in CO_2_ (decrease in a and b twice in the morning and in the afternoon) are due to cartridge sampling (data is not shown). The sharp increase at 1:45 h after participants entered the chamber stems from moving and stretching for 10 min.

**Figure 2:**
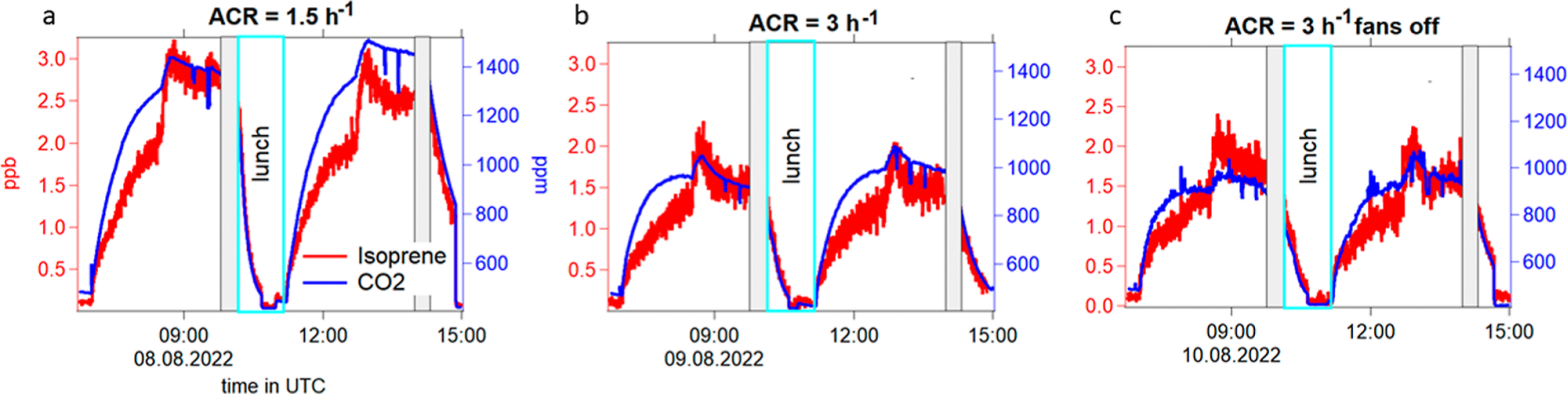
Time series of isoprene and CO_2_ with (a) air change rates of 1.5 h^–1^ and fans on; (b) air change rate of 3 h^–1^ and fans on; and (c) ACR of 3 h^–1^ and fans off. The *x*-axis is time in UTC; the left (red) axes show the isoprene mixing ratios in ppb; the right (blue) axes show CO_2_ VMRs. For better visualization, the axis labeling is only shown in (a) but is applicable for all other figures. The CO_2_ data was taken from the Picarro. The gray shaded areas were excluded from the analysis, as they were part of another experiment. The blue box indicates the lunch break.

While the primary breath emissions exhibit only subtle fan-related differences, oxygenated skin-oil products respond much more strongly. Figure 3 presents time-resolved VMRs of the two main oxygenated products originating from squalene ozonolysis, namely, 6-MHO and 4-OPA under varying air change rates and fan operations. In a total of 8 experiments, 6 experiments (3 + 3 replicates) investigated the effect of air change rates. Two of these, together with two additional experiments (2 + 2 replicates) investigated the influence of air mixing (ACR: 0.5 h^–1^ (a), 1.5 h^–1^ (b), and 3 h^–1^ (c), all with fans on, and 3 h^–1^ with fans off (d)). Their replicate experiments show similar mixing ratios and are shown in Supporting Information. When participants left the room for lunch (Figure 3b–d: 3:15–4:15) ozone increased due to an increase of outdoor ozone levels (leaking inside and not removed by filters; not actively injected) and people not reacting as an ozone loss. The small dips in 6-MHO and 4-OPA mixing ratios during lunch break stem from measuring supply air. As discussed before, for the ACR of 0.5 h^–1^, ozone was injected in the morning and participants stayed in the chamber for a consecutive time of 5 h, with a small bathroom break after 2.5 h for a duration of 10 min in which ozone was still injected.

**Figure 3:**
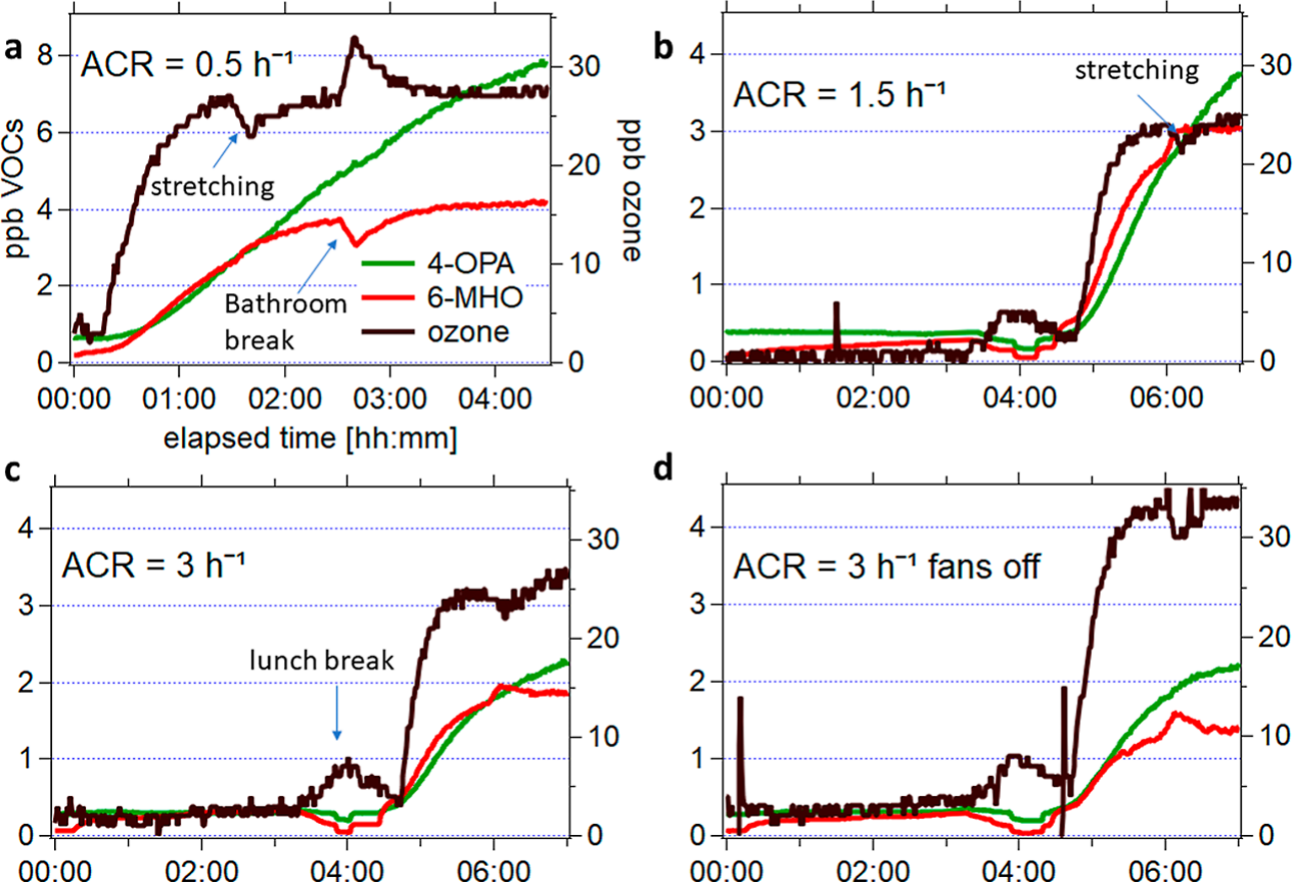
Time series of 6-MHO (C_8_H_15_O^+^) in red, 4-OPA (C_5_H_9_O_2_^+^) in green, and ozone in black shown for different ACRs and fan operations ((a) 0.5 h^–1^, (b) 1.5 h^–1^, and (c) 3 h^–1^, all with fans on and (d) 3 h^–1^ with fans off). Replicates for each experiment are shown in the Supporting Information (Figure S9). The *x*-axes show the elapsed time (in the format hh:mm), while the *y*-axes show the volume mixing ratio of the two oxygenated compounds. For better visualization, the axis labeling is only shown in (a) but is applicable for all other figures. Note that the *y*-axis range on (a) is different compared to the rest. Breath data is taken out and data points before and after breath are connected (2:50–3:15).

With an increase in the ACR from 0.5 to 3 h^–1^, a decrease in the VMRs of both primary and secondary VOCs was observed, confirming the dilution effect of ventilation. Interestingly, during the bathroom break highlighted in Figure 3a, 6-MHO decreased rapidly due to the removal of its human skin emission source, while 4-OPA continued to increase—albeit at a slightly reduced rate. This difference in temporal behavior can be explained as 4-OPA is a secondary product formed from the oxidation of 6-MHO, some of which remained in the air or on surfaces in the chamber. Concurrently, ozone levels rose during the break due to reduced surface loss, as the absence of occupants decreased ozone loss reactions. When participants moved during the exercise break (for 10 min) highlighted in Figure 3a,b (but also visible in c and d), a very small increase in 6-MHO VMRs was observed, accompanied by a decrease of ozone VMRs. This indicated that movement enhanced ozone consumption via surface reactions, likely on skin and clothing.

Under the fans-off condition (Figure 3d, ACR = 3 h^–1^), greater temporal variability in VMRs was observed, indicating less homogeneous air mixing. Despite an identical ozone input, higher steady-state ozone levels were measured without fans, which can be explained by reduced air movement over surfaces (walls, floor, roof, and people), decreasing ozone deposition rates. Both 6-MHO and 4-OPA concentrations were lower under fans off conditions. This suggests that the presence of operating fans enhanced air movement and increased air-to-skin mass transfer of ozone and thus 6-MHO generation rates. Although ozone is more abundant in the fans-off case, the lowered 6-MHO emission leads to a slightly reduced formation of 4-OPA (almost reaching steady state at the end of the experiment with fans off but not with fans on), which is derived from 6-MHO oxidation among other sources. However, it should be noted that GA is another squalene ozonolysis product that can generate both 6-MHO and 4-OPA. Salvador et al.^30^ demonstrated through a series of mass balance models the complexity of the interaction between ozone, GA, 6-MHO, and 4-OPA as well as ventilation. However, we consider that a likely explanation of the relatively small change in 4-OPA levels between fans-off and fans-on conditions is a more efficient oxidation of 6-MHO in the gas phase due to higher levels of ozone. In other words, higher ozone might compensate for lower 6-MHO available for the production of 4-OPA. Still, in order to understand the formation in detail, a model, simulating the temporal and spatial distribution as well as the kinetics, is necessary.

To further investigate the influence of ACR on oxidized volatile organic compounds (Figure 4a,b), VMRs were calculated for a 10 min period from 2 h 20 min after occupants entered the chamber in the morning (low ACR) or after lunch break with ozone present (two higher ACRs; specific times in the Supporting Information). We used these standardized time periods instead of the steady-state values for consistency because not all compounds reached steady state at the end of the experiment. Only oxidized compounds were included in this analysis, as data for the lowest ACR under nonozonated conditions were unavailable. To enable a direct comparison, background-subtracted VMRs (unoccupied chamber in the morning, specific times in the Supporting Information) were normalized to the highest mean value observed across all ACRs, which was always found at the lowest ACR. CO_2_ was also plotted for reference, as it is commonly used as a proxy for ventilation and human occupancy. For CO_2_ mixing ratios for the lowest ACR, only the data from the replicate experiment was used, as contamination occurred during the first run due to a not fully closed valve after taking cartridge samples (this did not influence VOC data; see Figure 1).

**Figure 4:**
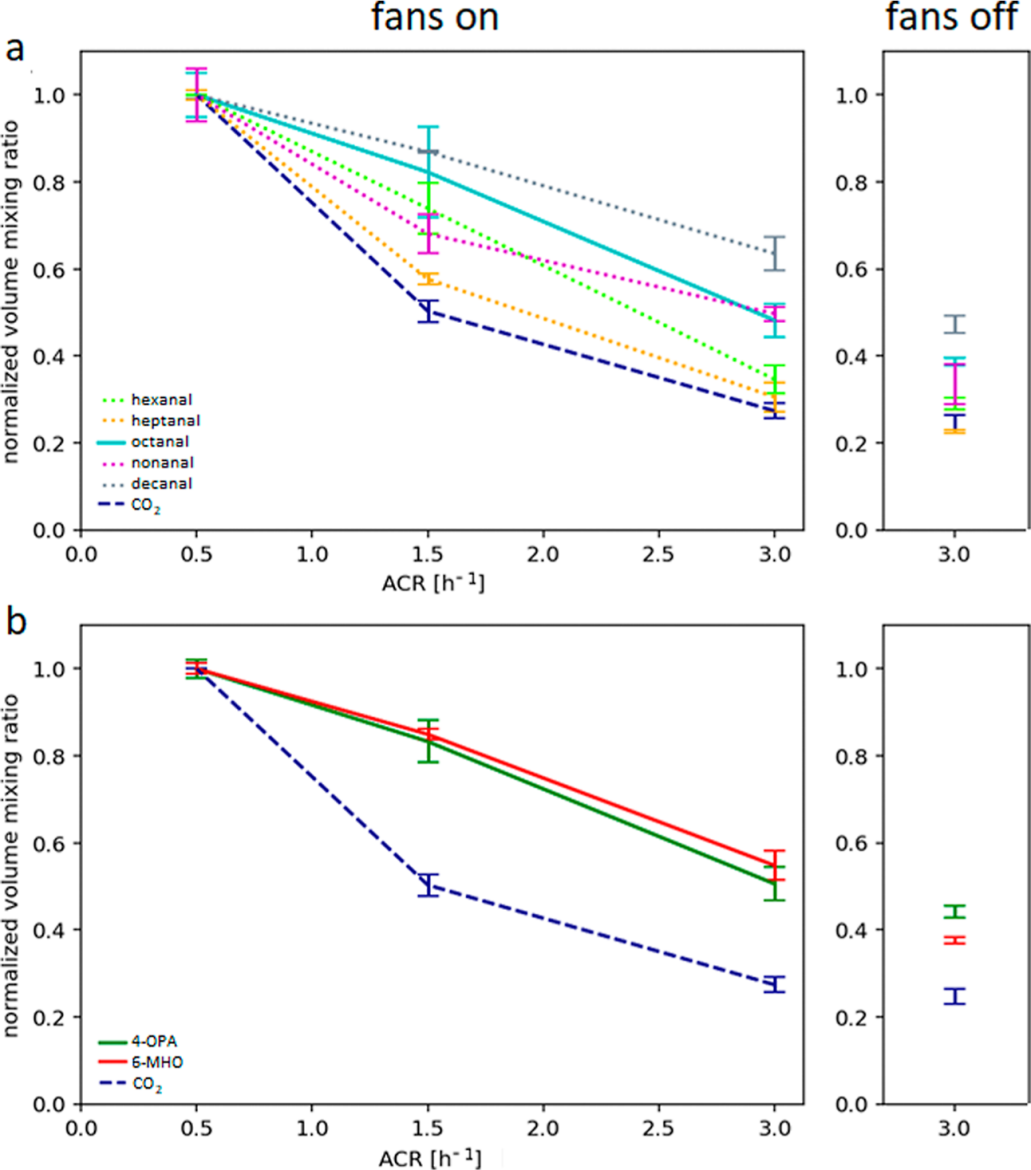
Normalized volume mixing ratios of a) C6-C10 aldehydes and b) 4-OPA and 6-MHO compared to CO_2_ as a function of the air change rate (left) and a comparison to fans off (right) calculated for the period 2:20–2:30 h after participants entered the chamber. The morning background taken before the participants entered the chamber was subtracted. This background-corrected VMRs were then normalized against the mean of the VMR at 0.5 h^–1^ (highest values). The bars show the calculated normalized VMRs, the lines are made from their mean values. Compounds measured by Vocus are shown as continuous lines. The dotted lines are data points from the NO^+^-CIMS. CO_2_ is shown as a dashed line.

Figure 4a shows the response of aldehydes —formed via ozonolysis of unsaturated fatty acids on the human skin—and of CO_2_ to changes in ACR. In addition, the fans-off scenario is added on the right. As already observed for 6-MHO and 4-OPA, the aldehyde levels decrease with less air mixing in comparison to the same ACR with fans on. Other results revealed by this figure are variabilities in the response of aldehydes to increasing ACR (note that the indicated time frame does not show steady-state conditions). As CO_2_ only has one source (human breath) and one sink (ventilation) in the chamber, a difference for chemicals produced through ozonolysis compared to CO_2_ was to be expected. However, how big the differences on changes in ACR are was not known. While CO_2_ VMRs decreased by approximately 50% when ACR increased from 0.5 h^–1^ to 1.5 h^–1^, a similar reduction in decanal required an ACR increase to above 3 h^–1^. This indicates that decanal is less responsive to ventilation than CO_2_, likely due to its lower volatility (higher molecular weight and longer CH_2_ chain) and greater potential for partitioning into surface layers or adsorption on indoor materials. With an increase in ACR, this effect is lower, and even desorption might occur, due to lower decanal levels in the gas-phase. Also, additional sources and sinks (such as reactions with the OH-radicals) cannot be ruled out.

The same effect can be seen for nonanal; however, relative nonanal concentrations, nonanal being more volatile than decanal—are lower with increasing ACR than those of decanal.

The different slopes for octanal (C_8_H_15_^+^—used for this plot, being the main fragment with a higher signal compared to the parent ion) can be explained as the data were derived from the Vocus, which is incapable of distinguishing carbonyl functional groups. Therefore, other compounds, such as ketones or alcohols that might stem from different sources than human skin oxidation, can alter the trend. As heptanal is more volatile than nonanal and decanal, it was expected to show a more pronounced effect on a change in ACR. Counterintuitively, it showed a stronger response to a change in ACR (similar to that of CO_2_) than the less volatile hexanal. This might be explained by a small influence from 4-OPA on the signal of hexanal, which cannot be ruled out, despite, the time series showing clear differences. An influence of 4-OPA on the hexanal signal could thus show a trend that is more similar to 4-OPA. Another possible explanation could be the differences in solubility: the more CH_2_ moieties present in the chains, the lower the solubility in water due to the molecule being less polar. Hexanal is more soluble in water than heptanal and may partition into the thin aqueous film present on all indoor surfaces, where it can be temporarily stored and released slowly, leading to a delayed response to ventilation. Heptanal, while less volatile than hexanal, may not be sufficiently soluble to partition into water films and instead remains in the gas phase, making it more directly responsive to dilution. However, in this study, the humidity was kept stable during the experiments, with 50 ± 5% measured at the desk. Future studies with varying humidities are necessary to further investigate this behavior. The flushing rates of organic compounds through indoor spaces, relative to that of CO_2_, are a complex function of the physical properties of the specific molecules, including both molecular weight and solubility.

Figure 4b further illustrates the effect of ACR on the mixing ratios of 6-MHO, 4-OPA and CO_2_. Again, CO_2_ shows a much stronger drop when increasing ACR, than 6-MHO and 4-OPA. With an increase from an ACR of 0.5 h^–1^ to 3 h^–1^, the VMRs of 6-MHO and 4-OPA decrease to 50–60%.

## Discussion

CO_2_ is widely used as a conventional proxy for indoor air quality because it is human-associated, nonreactive, and inexpensive to measure. However, the results of this study demonstrate that many airborne pollutants do not track CO_2_. In particular, divergence from the behavior exhibited by CO_2_ with changes in ACR can be seen for a homologous series of aldehydes as well as for 6-MHO and 4-OPA (Figure 4). The main source of 6-MHO is the formation on the skin surface (Salvador et al.^30^ showed ∼70% formed on surfaces). The gas-phase production through a reaction of GA with ozone is lower but not negligible (accounting for ∼30%^30^). An additional result of their study was that most of the produced 4-OPA stems from the production on surfaces, with additional sources being the oxidation in the gas phase of 6-MHO and GA. However, it is important to note that in our study, we used human beings rather than T-shirts as chemical sources, so additional air movement and surface volatilization was instigated through the participant’s body heat. Under low ACR and fans-off conditions, weak convective flows associated with the body heat of the participant dominate^36^—air rises over occupants and descends along chamber walls. This creates localized zones of low ozone and a high VOC concentration near the body (often referred to as the personal cloud effect). When fans are switched on, this stratified flow is disrupted, increasing turbulence and mixing; however, gradients near a person might still be formed. Qu et al.,^27^ for example, estimated the surface yield of GA as 3% and of 6-MHO as 9.4%, compared to a much lower surface yield for 4-OPA of 0.27% (with an error of 3%). Due to both the relative low surface yield of 4-OPA reported in Qu et al.^27^ and participants being warmer than just shirts, the formation of 4-OPA on surfaces might be lower compared to those reported for t-shirts in Salvador et al.^30^

For aldehydes, although having the same production mechanism (formed via ozonolysis of skin-emitted unsaturated fatty acids),^27,28^ the effect is different from compound to compound. Heavier, less volatile aldehydes (nonanal and decanal) show a weaker response to a change in ACR. The effect on heptanal was the strongest, even though it is less volatile than hexanal. Hexanal is more soluble in water. Thus, hexanal might be absorbed into a thin water layer on the chamber surfaces and consequently retarded to a greater extent, therefore showing a different response on dilution (by increasing ACR). These observations show that the volatility, solubility, and surface partitioning of the specific chemicals jointly modulate the simple dilution effects of the ACR.

In contrast, CO_2_ being highly volatile and nonreactive responds rapidly and predictably to ventilation. To achieve the same reduction of decanal, 6-MHO, and 4-OPA, the ACR had to be more than doubled (ACR of 1.5 h^–1^: ∼50% decrease in CO_2_ mixing ratio; ACR of 3.0 h^–1^ < 50% decrease in decanal, 4-OPA and 6-MHO mixing ratios). This difference shows that CO_2_ cannot be considered a reliable indicator of indoor air pollution dynamics, particularly when ozone is present, reactive chemistry occurs, and VOCs are not in steady-state conditions.

Fan operation primarily altered the mixing and surface interaction rates. With fans off, a less homogeneous mixing was observed (Figure 2) and ozone mixing ratios rose (Figure 3), consistent with decreased air movement lowering surface losses. Under fans-on conditions, 6-MHO and 4-OPA VMRs increased (Figure 3). The net effect of air mixing on 4-OPA was modest because two drivers partially compete: higher ozone (fans off) versus higher 6-MHO (fans on). Under fans-off conditions, higher ozone levels would promote the formation of 4-OPA; however, the associated reduction in 6-MHO emission rates dampens this effect.

The spatial dynamics of air movement help explain these outcomes. When fans are switched on, the personal cloud effect gets disrupted, increasing turbulence and air mixing. This enhances the delivery of ozone to the skin surface, boosting 6-MHO production, while also distributing 6-MHO more uniformly, increasing its availability. These processes likely produce steep, transient microenvironment gradients around the body and in the breathing zone that remain unresolved by room-average sampling;^37^ targeted measurements and simulations could quantify these gradients and their exposure implications.

Studies have suggested the benefits of using fans for thermal comfort in the summer season and hot environments.^38–40^ These conditions often coincide with elevated ozone levels. Our results indicate that the benefits of indoor air quality and exposure depend on which pollutant poses the greater health risk. If ozone is more harmful than the sum total of oxidized products, it could be beneficial to suppress indoor ozone levels by maximizing surface losses using fans. If the suite of secondary products is more hazardous, then the use of fans for indoor air mixing would not be recommended. Currently, the human health impact of the oxidation products is not well documented. One study showed a worsening on pulmonary effects on children with asthma when ozone reaction products increase.^41^ However, in their study, PM_2.5_ also increased with an increase of ozonolysis products; thus it is unclear, whether the effect stems from oxidation products or PM_2.5_. Weschler and Nazaroff^42^ also linked ozone loss to adverse health effects; however, they also assumed a linear increase of secondary organic aerosol (SOA) formation with ozone loss. Another study investigated the influence of ozone reaction products on human health and showed a correlation to worsened lung functions. However, the measured effects were below the accuracy range of their used instrument, thus further studies are required.^43^ Concluding, it is not yet understood if ozonolysis products alone have an influence on human health; however, SOA formation triggered through ozonolysis seems to have an effect. Calculations of human premature mortality due to outdoor pollution are often based on PM_2.5_ and ozone with particles having a larger attributable factor in pollution-related deaths.^44–46^ The use of fans has the potential to reduce indoor ozone and particle concentrations (at least for ultrafine particle).^9^

These findings have important implications for both future research on indoor air and practical applications. For experimental design, the decision to use fans must be made consciously and justified based on the research question—fan operation is a deliberate intervention that alters chemistry, mixing, production rates of oxidized products, and exposure. Therefore, reporting ACR, fan status, and ozone concentrations explicitly—and standardizing them when comparing studies—is essential. For real-world applications, this study reinforces that higher ACRs are favorable for reducing indoor-originating air pollutants, especially if the introduction of more ozone and other outdoor pollutants into indoor environments can be prevented.

In summary, this work reveals that indoor air quality is governed by a dynamic interplay among ventilation, air mixing, surface interactions, and reactive chemistry. The assumption that low CO_2_ values alone are an indicator for good air quality is oversimplified. Fan use not only homogenizes chemical gradients but also impacts pollutant concentrations by altering mass transfer and surface loss. Future research should focus on evidence-based ventilation strategies and should pair higher ventilation rates with explicit consideration of air mixing, guided by high-resolution spatial modeling and real-time measurements of both gas- and particle-phase chemistry. Furthermore, the chronic effects of key oxidation products—relative to ozone—require clarification to support risk-based indoor air quality guidelines.

## Supplementary Material



## Notes

Calculation of the ACR; instrumental conditions from Vocus; used calibration procedure and its outcome from Vocus and the NO^+^-CIMS; potential influence on the hexanal signal from both CIMS; results from the replicate runs underlying Figure 3 of the paper; timeframes used for the analysis and a discussion on the used time frame for Figure 4 of the paper; and more detailed discussion on the fragmentation of aldehydes within the Vocus and how the fragmentation was tested (PDF)
