# Supplementary material for: Influence of Ventilation Rate and Indoor Air Mixing on Ozone–Human Skin Chemistry
Source: ACS EST Air. 2026 Mar 30;3(4):1046–56. doi: 10.1021/acsestair.5c00433 (PMC13077628; doi:10.1021/acsestair.5c00433)
Supplement: Supplementary file 1 [file ea5c00433_si_001.pdf]

Supporting Information for

Influence of ventilation rate and indoor air mixing on ozone–human skin chemistry

Tatjana Arnoldi-Meadows[1], Nijing Wang[1], Gabriel Bekö[2], Marouane Merizak[3], Pawel Wargocki[2], Meixia Zhang[3,4], Shen Yang[3,5], Dusan Licina[3], Jonathan Williams[1,6,*]

Affiliations

[1]Max Planck Institute for Chemistry, Hahn-Meitner Weg 1, 55128 Mainz, Germany
[2]International Centre for Indoor Environment and Energy, Department of Environmental and Resource Engineering, Technical University of Denmark, 2800 Kongens Lyngby, Denmark.
[3]Human-Oriented Built Environment Lab, School of Architecture, Civil and Environmental Engineering, École Polytechnique Fédérale de Lausanne (EPFL), 1015 Lausanne, Switzerland
[4]Beijing Institute of Technology, School of Mechanical Engineering, Beijing, CN 100081
[5]School of Architecture, Southeast University, 210096 Nanjing, China
[6]Climate and Atmosphere Research Center, The Cyprus Institute, 1645, Nicosia, Cyprus

*Corresponding author: Email: jonathan.williams@mpic.de

Total number of pages: 24
Total number of figures: 12
Total number of tables: 7

This Supporting Information file includes:

Section S1. Calculation of ACR 3
Section S2. Instrumental conditions Vocus 4
Section S3. Calibration procedure 4
Section S4. Outcome calibration: Vocus 5
Section S5. Outcome calibration NO+-CIMS 12
Section S6. Potential influence on hexanal signal 13
Section S7. Replicates of figure 3 18
Section S8. Specific times used for analysis 19
Discussion on using ACR/vmr correlation time frame 19
Section S9. Fragmentation tests of Aldehydes 21

Figure S1 k-rates to sensitivity correlation for first week's calibration. 10
Figure S2 Transmission curve for first week's calibration 10
Figure S3 k-rate to sensitivity correlation for second week's calibration. 11
Figure S4 Transmission curve for second week's calibration 11
Figure S5 Influence of 4-OPA on hexanal signal for Vocus. 14

Figure S6 Comparison between hexanal signal from Vocus and NO^+-CIMS. 15
Figure S7 Mass spectrum 4-OPA and hexanal from NO^+-CIMS 16
Figure S8 Time series and scatterplot for 4-OPA and Hexanal measured by the NO^+-CIMS. 17
Figure S9 replicates of figure 3 in the main paper... 18
Figure S10 relationship ACR/VMR near steady state. 20
Figure S11 setup scheme for aldehyde tests. 22
Figure S12 Time series of aldehyde fragmentation tests for the Vocus instrument 22

Table S1 Calibration output Vocus 06.08.2022 used for measurements during the first week 6
Table S2 Calibration output Vocus 18.08.2022 used for measurements during the second week 7
Table S3 Calibration output Vocus for 4-OPA and 6-MHO: used for whole experiment 8
Table S4 Calibration results NO^+-CIMS 12
Table S5 Specific times in UTC used in analysis 19
Table S6 Main fragments of reported aldehydes measured by Vocus and used multiplication factor for aldehyde quantification. 23
Table S7 Major fragments of reported aldehydes measured by NO^+-CIMS 23

Section S1. Calculation of ACR

Air changes were calculated from the exponential decrease in CO_2 volume mixing ratios (VMR) after participants exited the chamber, using the flowing equation. To be consistent with previous work the CO_2 data was taken from the Li-COR 850 CO_2 monitor.

$$N = \frac{\left[\ln\left(C_{\mathrm{int}}^{t_0} - C_{\mathrm{ext}}\right) - \ln\left(C_{\mathrm{int}}^{t_1} - C_{\mathrm{ext}}\right)\right]}{t_1 - t_0}$$

with

N = number of air changes per hour

$C_{\mathrm{int}}^{t_0}$ = internal CO_2 concentration at the time participants left the chamber

C_{ext} = external CO_2 concentration in the supply air

$C_{\mathrm{int}}^{t_1}$ = internal CO_2 concentration at the end of the decay period

t_0 = time when participants left the chamber (in h)

t_1 = time at the end of the decay period (in h)

Section S2. Instrumental conditions Vocus

The Vocus ran under following conditions: water flow (primary ion): 20 sccm, IMR pressure: 2 mbar, reactor temperature: 60°C, drift voltage: 475 V, IMR amplifier: 400 V, Ion Lens 2 voltage: -160 V, deflector voltage: 156.9 V, deflector flange voltage: -161.65 V, Skimmer 2: 11.6 V, reference Bias voltage: -14.4 V, BSQ front: -8.04 V, BSQ back: -11.7 V, BSQ voltage: 275 V, Skimmer: 1.4 V, Lens Skimmer: 0 V, Nozzle: 0 V. Resulting in an E/N of 110 Td.

Section S3. Calibration procedure

Calibrations were performed using a gas standard containing 18 volatile organic compounds (VOCs) (Apel-Riemer Environmental Inc., USA). A step wise calibration with varying volume mixing ratios was performed by precisely controlling gas flows using mass flow controllers (MFCs). One MFC regulated the flow of the calibration standard, while two additional MFCs were used to control the dilution flows—one for dry air and one for humidified air (wet air). For each compound, the known volume mixing ratio (in ppb) was plotted against the instrument signal (in ions/s), and the resulting linear regression was used to determine the instrument sensitivity (ions/s per ppb). During the experiment, the measured signal was divided by this sensitivity to calculate the real-time volume mixing ratio of each VOC.

The Limit of Detection (LoD) was determined as three times the standard deviation of the signal measured during zero-air measurements (performed during each calibration), divided by the instrument sensitivity. This yields the LoD in ppb, representing the lowest concentration reliably distinguishable from background noise.

Section S4. Outcome calibration: Vocus

Calibrations were performed twice during the experimental time frame. For Vocus, the output of the calibration from 06.08.2022 (Table S1) was applied for the first week; for the second week the calibration output from 18.08.2022 (Table S2) was applied.

Relative uncertainties in the calculated volume mixing ratios were estimated by combining contributions from different sources:

(1) statistical uncertainty from
  a) flow measurements
  b) standard deviation from instrument’s signal
(2) systematic uncertainties from
  a) calibration standard mixing ratio
  b) flow measurement device
  c) slope of the calibration curve
  d) systematic error due to software’s failure to subtract the background signal before sensitivity calculation

Only the possible systematic error due to the instrument software’s failure to subtract the background signal before sensitivity calculation, could be prevented. All other uncertainties cannot be reduced by the experimenter with the given setup.
This additional uncertainty, in principle, could introduce bias if the background signal was not stable over time. However, only the error for acetone exceeded 5% in both performed calibrations. As the error was determined to be negligible, a repetition of the analysis was not performed. Still, the error is represented in the total systematic uncertainty in Table S1 and Table S2.

Table S1 Calibration output Vocus 06.08.2022 used for measurements during the first week, first column: compound names; second column: sensitivity; third column: relative statistical error including uncertainties of flows, and standard deviation of Vocus' signal; fourth column: relative systematic uncertainty: uncertainty from flow measurement device, uncertainty of calibration slope, and uncertainty due to shifting background ; fifth column LoD calculated by 3σ of zero air measurement.

Compounds in calibration standard	Sensitivity [(ions/s)/ppb]	Relative Statistical uncertainty<br><br>flows + Vocus signal	Relative systematic uncertainty<br><br>Flow measurement + slope uncertainty + uncertainty due to shifting background	Limit of Detection (=LoD) [ppt]
Acetone	5180	< 1 %	< 20 %	97
Acrylonitrile	3070	< 1 %	< 10 %	1.6
Isoprene	578	< 5 %	< 10 %	39.5
DMS	2110	< 5 %	< 10 %	4.5
MVK	4020	< 5 %	< 10 %	7.4
MEK	4430	< 5 %	< 10 %	8.6
Benzene	162	< 5 %	< 10 %	59.8
m-Xylene	1590	< 5 %	< 10 %	51.8
α-Pinene	873	< 5 %	< 10 %	7.9
TMB	1760	< 5 %	< 10 %	3.4
D4	1770	< 5 %	< 10 %	2.7
D5	1520	< 5 %	< 10 %	1.1
β-Caryophyllene	978	< 5 %	< 10 %	3.3

Table S2 Calibration output Vocus 18.08.2022 used for measurements during the first week, first column: compound names; second column: sensitivity; third column: relative statistical error including uncertainties of flows, and standard deviation of Vocus' signal; fourth column: relative systematic uncertainty: uncertainty from flow measurement device, uncertainty of calibration slope, and uncertainty due to shifting background ; fifth column LoD calculated by 3σ of zero air measurement.

Compounds in calibration standard	Sensitivity (1/calibration factor) [(ions/s)/ppb]	Relative Statistical uncertainty<br><br>flows + Vocus signal	Relative systematic uncertainty<br><br>Flow measurement + slope uncertainty + uncertainty due to shifting background	Limit of Detection (=LoD) [ppt]
Acetone	4910	< 1 %	< 15 %	10.7
Acrylonitrile	3500	< 1 %	< 10 %	0.2
Isoprene	615	< 5 %	< 10 %	10.2
DMS	2310	< 5 %	< 10 %	1.1
MVK	4640	< 5 %	< 10 %	1.6
MEK	5000	< 5 %	< 10 %	1.7
Benzene	175	< 5 %	< 10 %	18.4
m-Xylene	1730	< 5 %	< 10 %	1.5
α-Pinene	959	< 5 %	< 10 %	2.0
TMB	1940	< 5 %	< 10 %	0.7
D4	1960	< 5 %	< 10 %	0.6
D5	1690	< 5 %	< 10 %	0.3
β-Caryophyllene	1100	< 5 %	< 10 %	0.7

Table S3 Calibration output Vocus for 4-OPA and 6-MHO: used for whole experiment first column: compound names; second column: sensitivity; third column: relative statistical error including uncertainties of flows, and standard deviation of Vocus' signal; fourth column: relative systematic uncertainty: uncertainty from flow measurement device, uncertainty from mass flow controller and uncertainty of calibration slope; fifth column LoD calculated by 3σ of zero air measurement.

Compounds in calibration standard	Sensitivity [(ions/s)/ppb]	Relative Statistical uncertainty<br><br>Vocus signal + estimated error of flow uncertainty of 1%	Relative systematic uncertainty<br><br>Flow + slope uncertainty + uncertainty due to shifting background	Limit of Detection (=LoD) [ppt]
4-OPA	3050	< 5 %	< 20 %	6
6-MHO	2830	< 5 %	< 20 %	13

Two fits are needed to calculate volume mixing ratios for compounds not included in the calibration standard.[1] The first is the linear correlation between sensitivity and k-rates (Figure S1 and Figure S3). The second, showing the dependency of the sensitivity (taken the k-rate to sensitivity correlation into account) on its mass to charge ratio, resulting in a sigmoidal curve (Figure S2 and Figure S4). The equation to calculate VMRs not included in the calibration standard is given by:

$$vmr[\text{ppb}] = \frac{signal\left[\frac{\text{ions}}{\text{s}}\right]}{linear\_corr(k) \cdot sigmoid(m)}$$

With: $signal\left[\frac{\text{ions}}{\text{s}}\right]$= the signal of the compound not included in the gas standard, linear_corr(k) = the linear correlation for the compound's k-rate

And sigmoid(m) = the output of the sigmoid fit at the mass to charge ratio of the compound.

To get those equations all fragments and isotopes need to be included from the compounds within the calibration standard. Thus, the sensitivities for some compounds on the following graphs (Figure S1 and Figure S3) might differ from the sensitivities shown in Table S1 and Table S2 (due to disregarding isotope and fragment signals for usual calibration).

Compounds that are lighter than m/z = 54 are excluded to fit the linear correlation between k-rate and sensitivity (Figure S1 and Figure S3). The k-rates were taken from Salthammer et al. 2023[2] (k=k_cap: E/N = 110 Td), for some compounds in our calibration standard no k-rates were published and therefore not included. Additionally, D4 and D5 were excluded, as these points did not seem reasonable. A possible explanation is a mismatch in the published k-rates.

For fitting the transmission curve, lighter ions are included (Figure S2 and Figure S4). Additionally, the base was fixed to “0”.

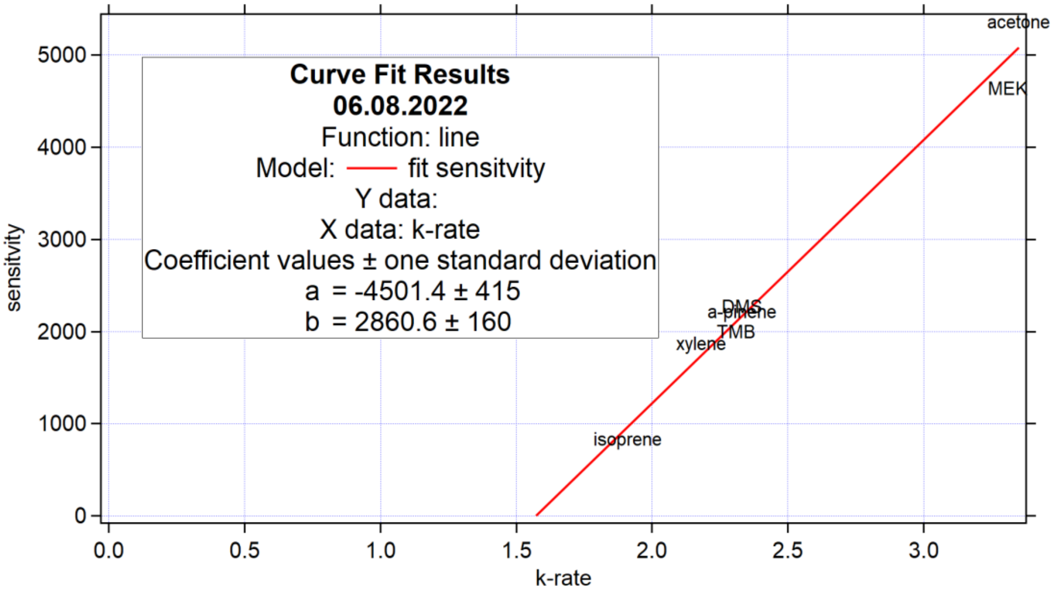

Figure S1 k-rates to sensitivity correlation for first week's calibration. The sensitivities from different compounds are plotted against their associated k-rates to see the linear correlation of both. The fit gives following equation: s = -4501.4 ± 415 + k(2860.6 ± 160); with s = sensitivity and k = k-rate for specific compounds. k-rates taken from Salthammer et al. 2023[2] (k=k_cap with an E/N of 110)*

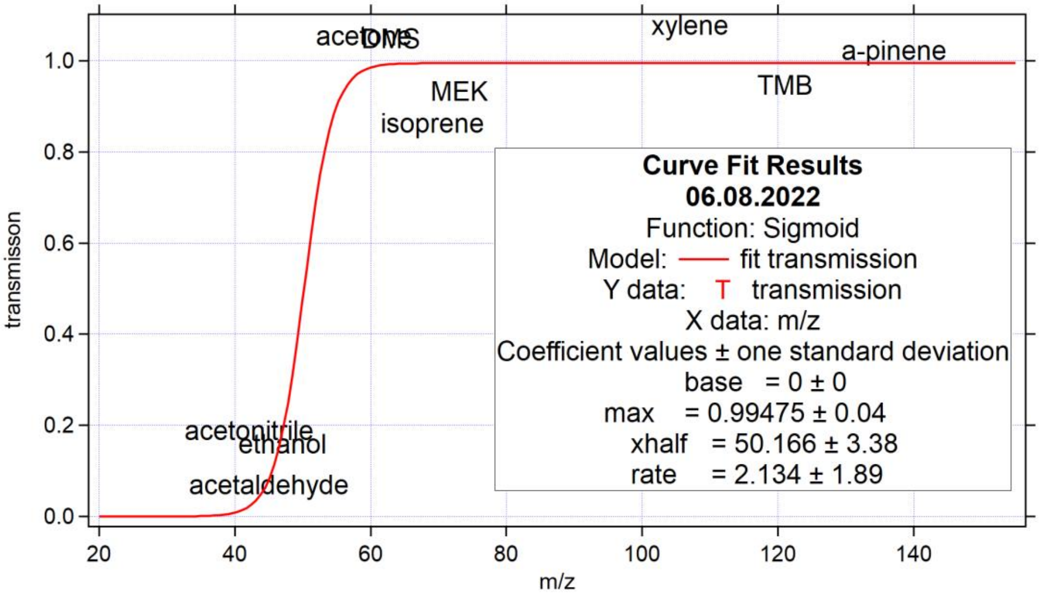

Figure S2 Transmission curve for first week's calibration: The y axis shows the measured sensitivity devided by the calculated sensitivity (with the equation from Figure S1)The x-axis shows the mass to charge ratio. The obtained sigmoidal curve has following parameters: base = 0 ± 0; max = 0.99475 ± 0.04; xhalf = 50.166 ± 3.38; rate = 2.134 ± 1.89

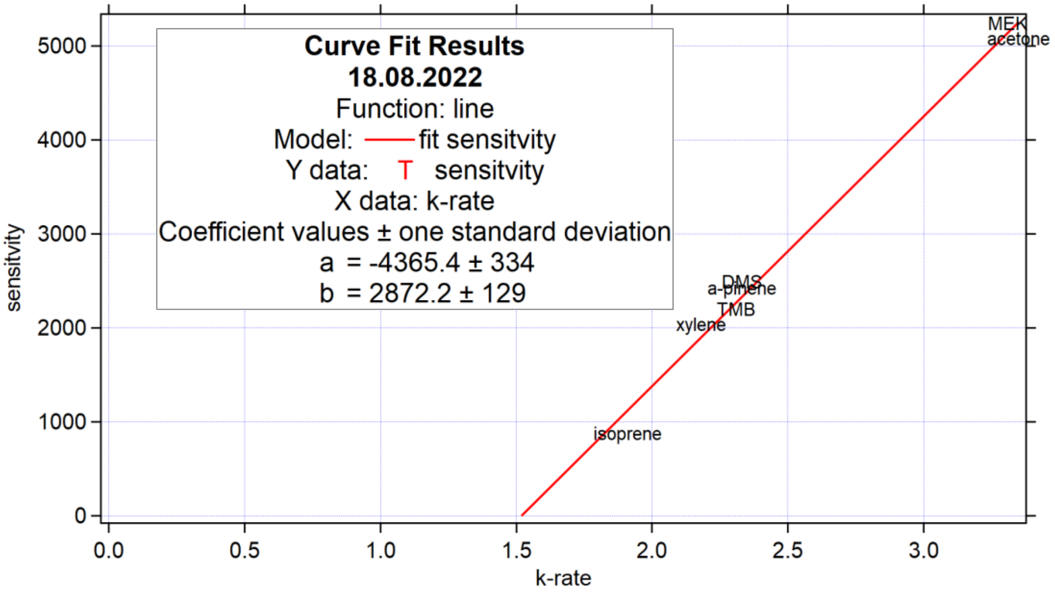

Figure S3 k-rate to sensitivity correlation for second week's calibration. The sensitivities from different compounds are plotted against their associated k-rates to see the linear correlation of both. The fit gives following equation: s = -4365.4 ± 334 + k(2872.2 ± 129); with s = sensitivity and k = k-rate for specific compounds. The k-rates were taken from Salthammer et al. 2023[2] (k=k_cap with an E/N of 110)*

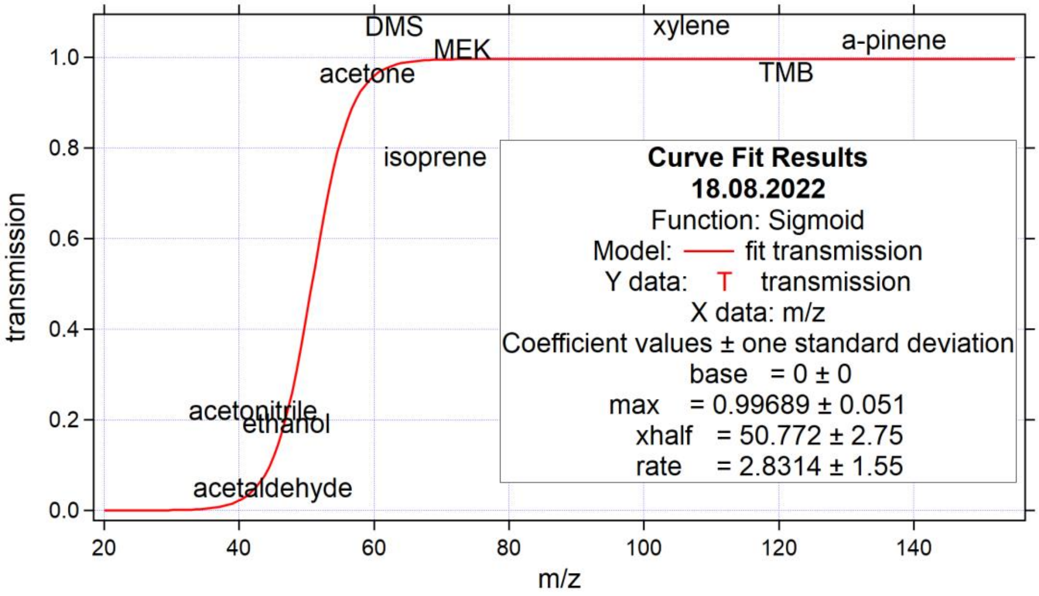

Figure S4 Transmission curve for second week's calibration: The y axis shows the measured sensitivity devided by the calculated sensitivity (with the equation from Figure S3). The x-axis shows the mass to charge ratio. The obtained sigmoidal curve has following parameters: base = 0 ± 0; max = 0.99689 ± 0.051; xhalf = 50.772 ± 2.75; rate = 2.8314 ± 1.55

Section S5. Outcome calibration NO^+-CIMS

For the NO^+-CIMS the same procedure was used for calibration resulting in the calibration outputs shown in Table S4

Table S4 Calibration results NO^+-CIMS; first column: compound names, second column: LoD calculated by 3σ of zero air measurement

Compound	Sensitivity [ncps/ppb]	Total uncertainty	LoD [ppt]
4-OPA	9.5	< 7%	110
6-MHO	7.2	< 6%	122
Acetaldehyde	1.7	< 29%	307
Ethanol	2.9	< 24%	133
Acetone	2.7	< 16%	143
DMS	10.9	< 11%	18
Isoprene	8.0	< 17%	15
MEK	6.0	< 12%	18
Benzene	6.9	< 14%	14
Xylene	10.9	< 12%	9
Trimethylbenzene	10.8	< 9%	14
a-Pinene	4.7	< 16%	13
b-Caryophyllene	3.0	< 18%	23
D4	4.0	< 14%	10

Section S6. Potential influence on hexanal signal

It should be noted that the mass resolutions of both instruments were high enough to distinguish between 4-OPA and hexanal (see Figure S5 and Figure S7) and thus, the behavior of the hexanal signal in the paper (Figure 4 in the main text) was not influenced by an overlap. For Vocus this is shown by a mass spectrum of relatively high concentrations of both, 4-OPA and hexanal. Additionally, the shape of the time series of those compounds are the same for the fragments but differ for the two different compounds (see Figure S5 lower graph). Also, the data from Vocus and the NO^+-CIMS are comparable (see Figure S6), only showing a discrepancy at higher ACRs. This discrepancy, however, might stem from other compounds on the exact same mass (the Vocus instrument can’t distinguish different carbonyls), which might stem from different sources than skin oil oxidation.

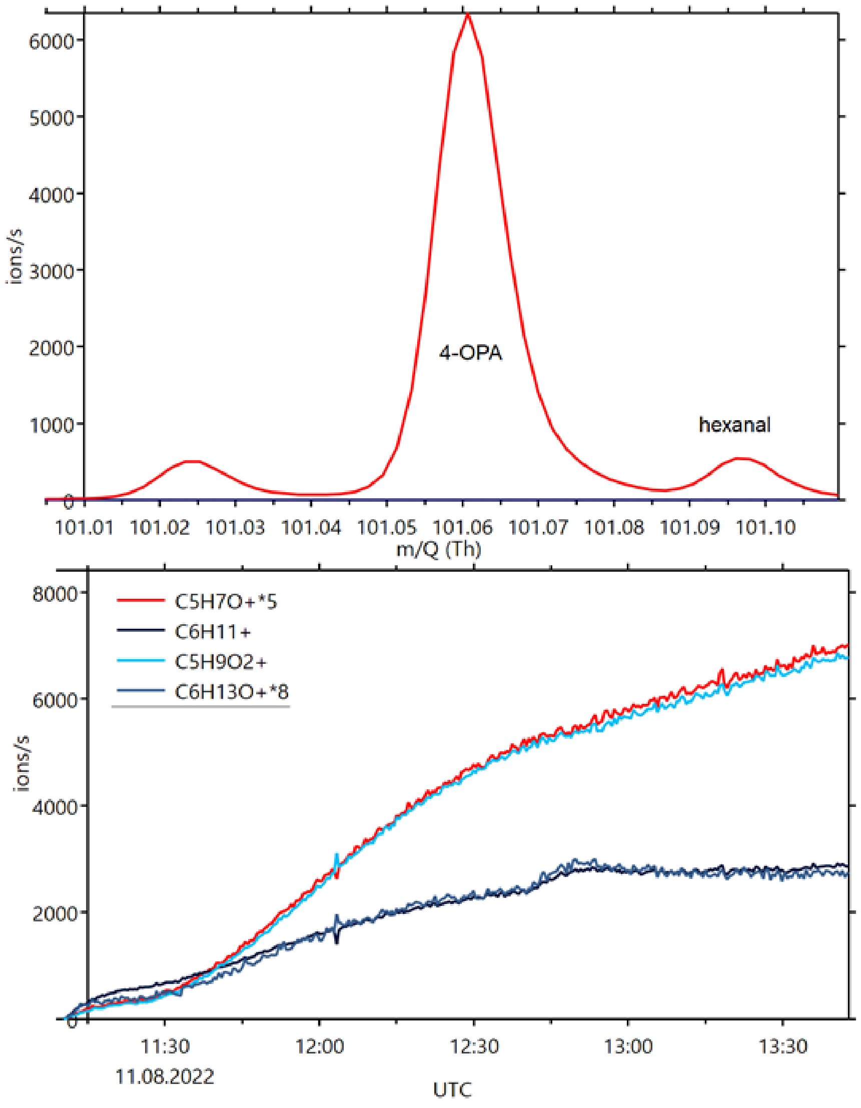

Figure S5 Influence of 4-OPA on hexanal signal for Vocus. Upper part: MS of last minutes of day 4; lower part: time series of 4-OPA, hexanal and their fragments from day 4 of the experiments.

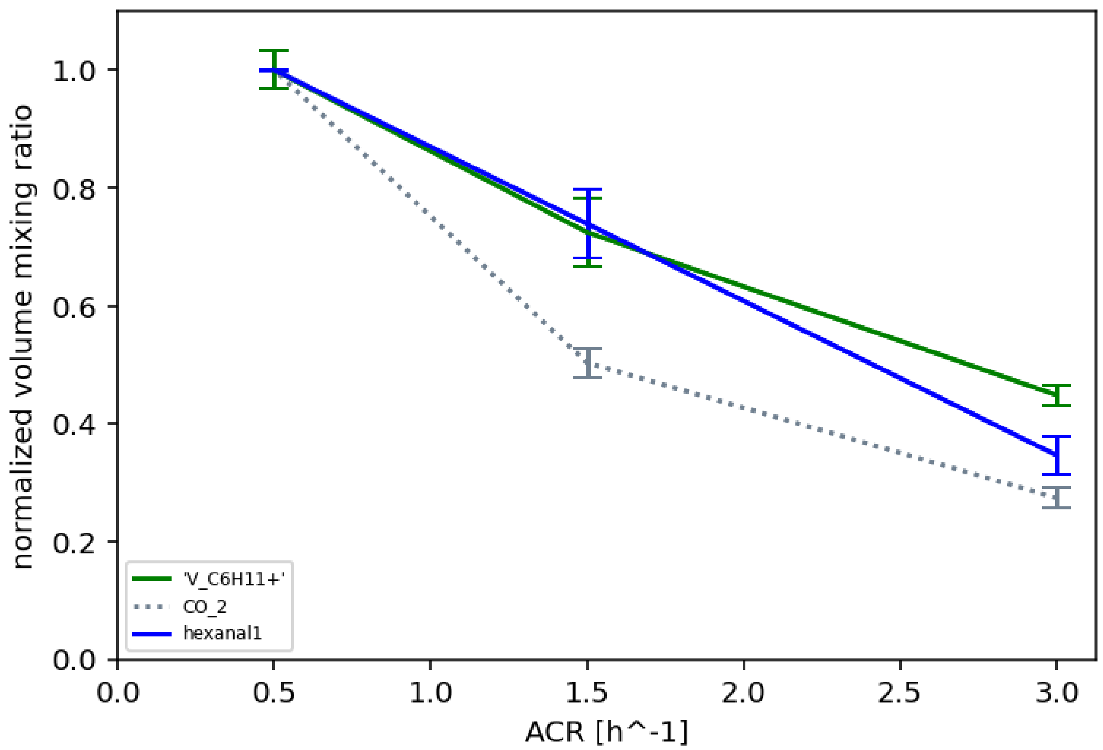

Figure S6 Comparison between hexanal signal from Vocus (in green) and NO^+-CIMS (in blue). Showing the same plot (with the exact same time frame) as in figure 4 of the main text.

For the NO^+-CIMS the mass resolution was also sufficient to distinguish the peaks from 4-OPA (m/z 99.044) and Hexanal (m/z 99.080), even though being in the shoulder of an isobaric ion shown in Figure S7. The used peak integration software (IDA) applies Cumulative Peak Analysis rather than multi-Gaussian fitting[3,4] This algorithm automatically detects the isobaric interference by analyzing the residuals of the primary peak fit, confirming the presence and location of other peaks. Meanwhile, unlike Gaussian fitting, where peak areas can float ambiguously during heavy overlap, cumulative analysis mathematically constrains the total signal intensity to the measured ion counts. Therefore, while the peaks are not baseline-separated, the high-precision fit faithfully attributes the signal "step" to the correct m/z channels with minimal interference error. In addition, a time series and a scatter plot of one experiment (3,0 h^{-1} air change rate, fans on) are shown in Figure S8 to better demonstrate the possibility of interference. The figure shows that hexanal reached steady-state condition after ozone injection around~ 1.5 to 2hr while 4-OPA kept increasing until the end of the experiment. The turning point shown in the inner scatter plot further confirms that during 4-OPA peak dominant period, hexanal can still be separately integrated. However, we do admit that small interference error still exists as those two peaks are not fully separated due to lower mass resolution compared to Vocus-PTR.

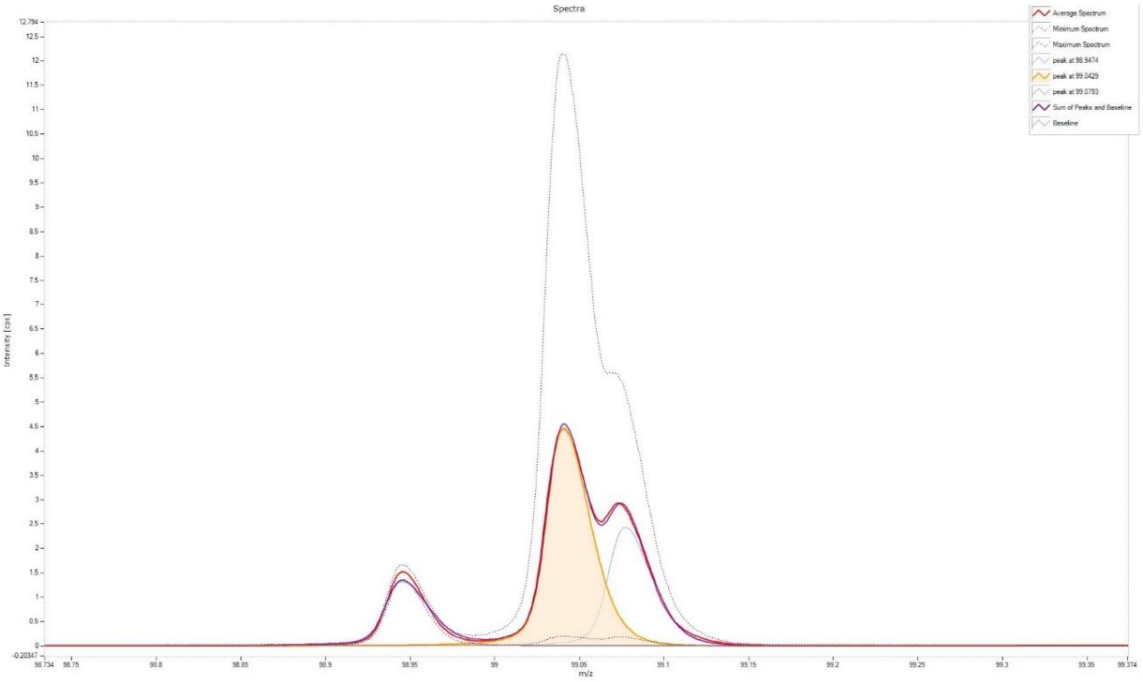

Figure S7 Mass spectrum 4-OPA and hexanal from NO^+-CIMS

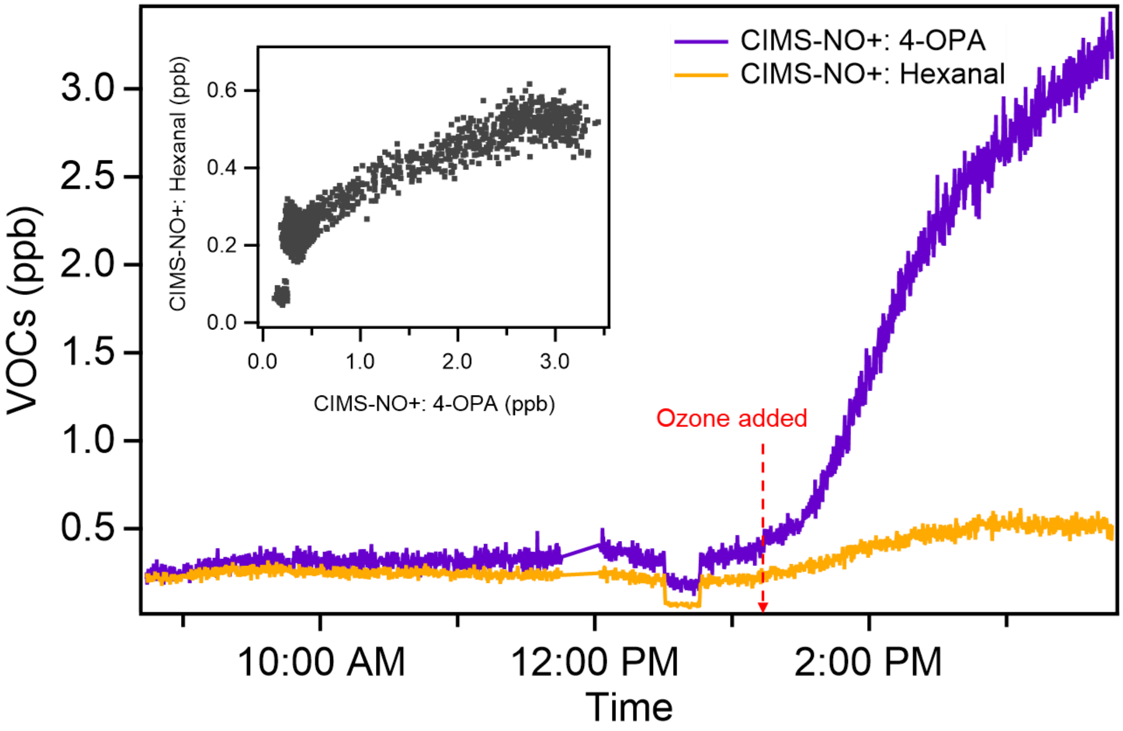

241

242 *Figure S8 Time series and scatterplot for 4-OPA and Hexanal measured by the NO^+-CIMS.*

Section S7. Replicates of figure 3

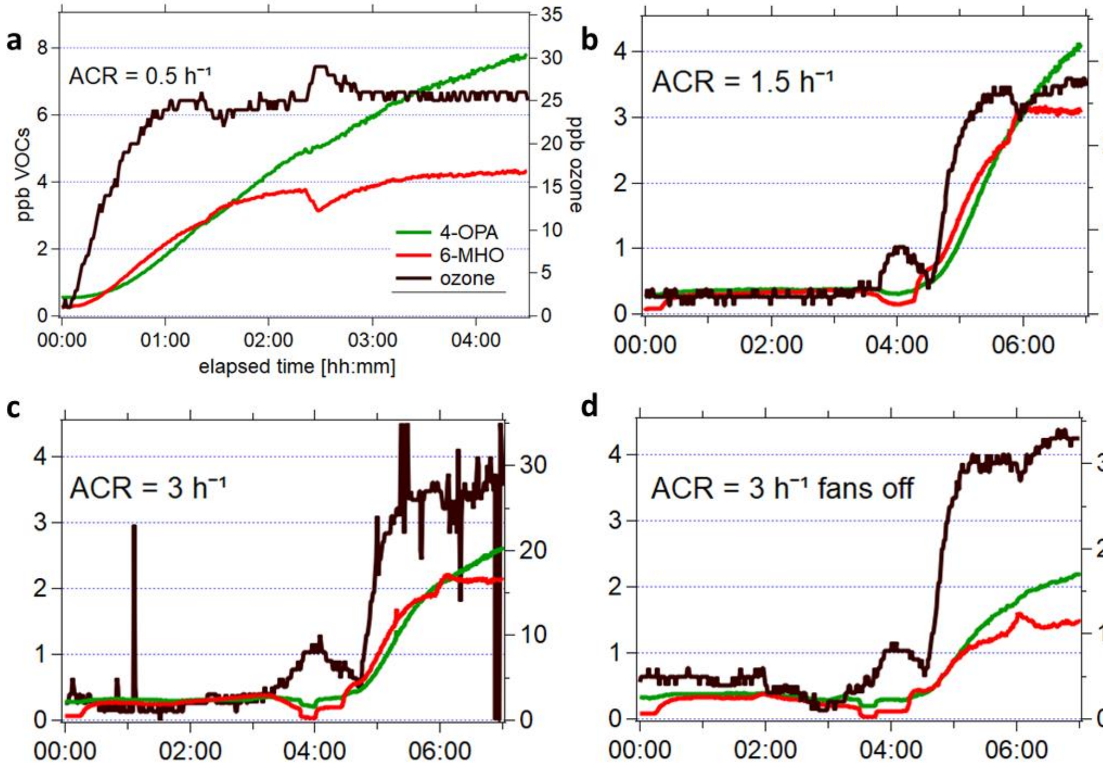

Figure S9 replicates of figure 3 in the main paper: Time series of 6-MHO ($C_8H_{15}O^+$) in red, 4-OPA ($C_5H_9O_2^+$) in green and ozone in black are shown for different ACRs and fan operations (a: 0.5 $^{-1}$, b: 1.5 h^{-1}, c: 3 h^{-1}, all with fans on and d 3 h^{-1} with fans off). The x-axes show the elapsed time, while the y-axes show the volume mixing ratio of the two oxygenated compounds. For better visualization the axis labeling is only shown ones in a) but is applicable for all other figures.

Section S8. Specific times used for analysis

Table S5 Specific times in UTC used in analysis

Date	ACR [h^{-1}] / fan status	Background (unoccupied chamber)	Instrument comparison	ACR/vmr correlation
08.08.2022	1.5 / on	06:40 – 06:55		13:31 – 13:41
09.08.2022	3 / on	06:40 – 06:55	13:40 – 13:50	13:30 – 13:40
10.08.2022	3 / off	06:40 – 06:55		13:30 – 13:40
11.08.2022	3 / on	06:45 – 06:55	13:40 – 13:50	13:30 – 13:40
12.08.2022	0.5 / on	07:35 – 07:50	12:35 – 12:45	10:20 – 10:30
15.08.2022	0.5 / on	07:35 – 07:50	12:20 – 12:30	10:10 – 10:20
16.08.2022	1.5 / on	05:45 – 06:00		13:20 – 13:30
17.08.2022	3 / off	06:40 – 06:55		13:20 – 13:30

Discussion on using ACR/vmr correlation time frame

The time frame used for the intercomparison of ACR/VMR was set to this specific time frame, as some reported VOCs (4-OPA for all ACRs but also C6-C10 aldehydes for the lowest ACR) did not reach steady state even after 4.5 hours for the lowest ACR. It should be noted that the graphs change if other time frames are used, due to the fact that the signal of the VOCs is in different transient phases. We tried to use data where the both higher ACRs are in or near steady state, but also wanted to use the data before participants left for a bathroom break during the low ACR experiments. In addition, this time frame was used to mimic a more realistic work day. At least in Germany a break from work is obligatory when working more than 8 hours. Thus, staying at one place to reach a steady state (without moving) is more unrealistic than the approach we decided on here. With those three ideas in mind (near steady state for higher ACRs, data before leaving for bathroom break, and unrealistic steady state in real life), the timeframe resulted in that provided above. Additionally, Figure S10 shows the same relationship between ACR and VMR for the last 10 minutes before breath measurements. The behavior shows a more similar trend to CO_2, except for 6-MHO. This might be explained by different sinks (further oxidation) compared to other VOCs. The convergence for the other VOCs to the CO_2 trend could have been expected, as the mass dependency

probably resulted from the compounds' stickiness and the related delayed achieved steady states.

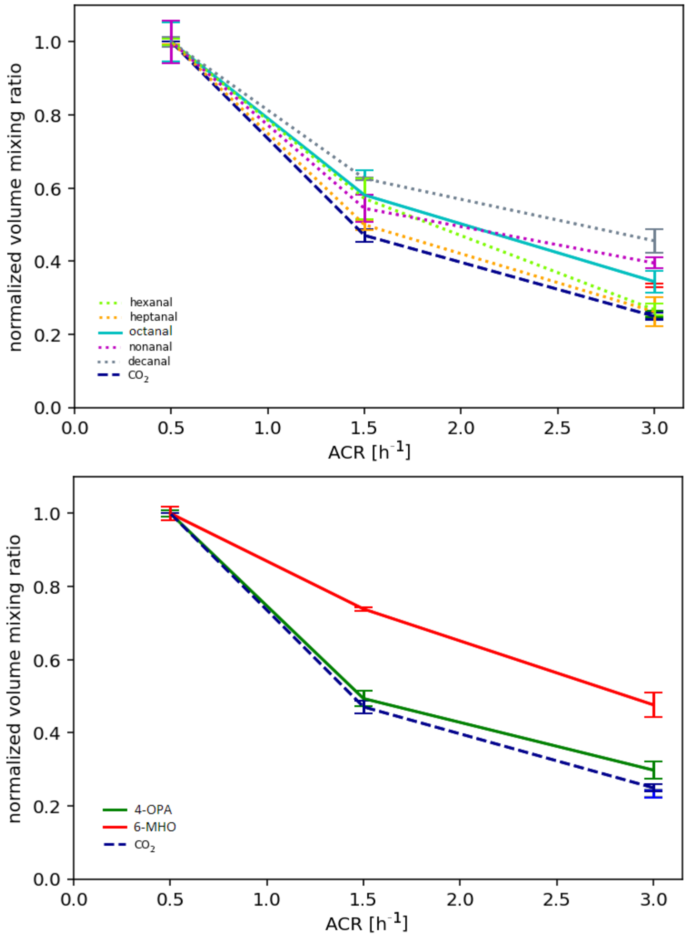

Figure S10 relationship ACR/VMR near steady state; same idea as in figure 4 in main text. Time frame changed to 10 minutes before breath measurements.

Section S9. Fragmentation tests of Aldehydes

As discussed in the paper, the settings used during the measurements produced considerable fragmentation, especially of aldehydes. In order to know the fragments occurring during our study, a test on the fragmentation of different aldehydes was performed in the same way as in Ernle et al. 2023.[5] Different aldehydes were injected in gaseous form (taken from the head space of the pure substance) with a syringe and using different carrier and dilution flows to adapt the signal height (see Figure S11). The different time series plotted in Figure S12 show the fragmentation patterns from C6-C9 aldehydes of Vocus, which were discussed in the paper. The test for decanal failed due to potential contamination of the decanal solution. However, the 4 other aldehydes already showed that fragmentation occurs inside the Vocus, and needs to be considered later in the data analysis. In this study we considered the ratio of the main fragment (aldehyde losing one water) from the aldehyde test and added this ratio to the signal of the parent mass. The contribution of fragments and the according multiplication factors are shown in Table S6. Note that the data from the aldehyde test here is not transmission curve corrected, thus, smaller ions have a bigger contribution to the overall signal. This leads to an underestimation, as only the main (heavier) fragments are included here.

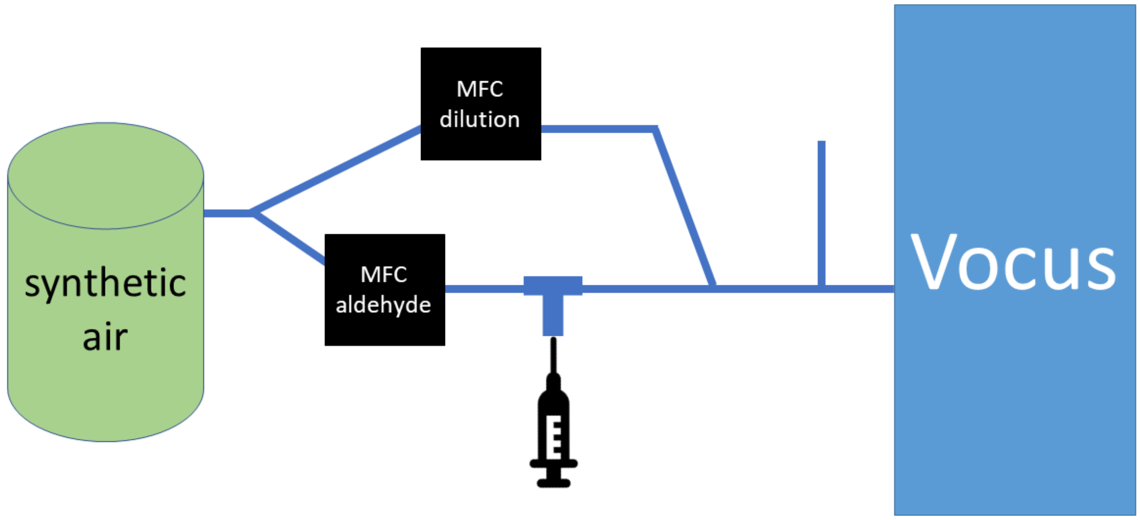

Figure S11 setup scheme for aldehyde tests. Synthetic air is used as a carrier flow (modified by MFC aldehyde) for aldehydes which are injected via a syringe and diluted with another synthetic air flow (MFC dilution).

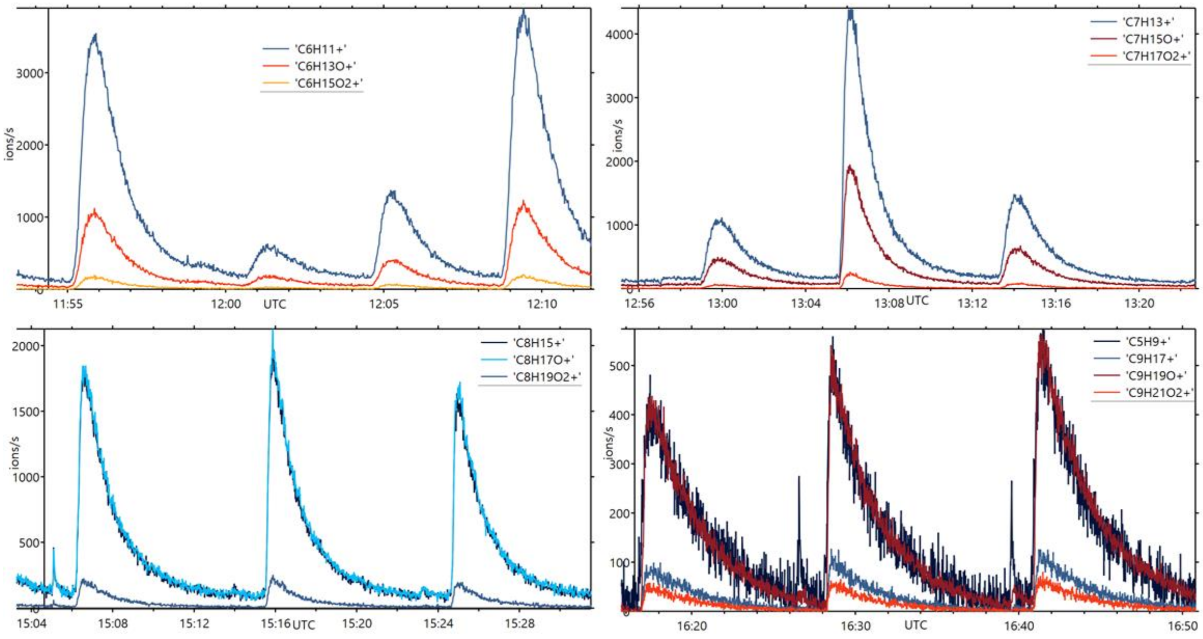

Figure S12 Time series of aldehyde fragmentation tests for the Vocus instrument. Upper left: hexanal, upper right: heptanal, bottom left: octanal and bottom right: nonanal. Shown are the fragments included in this manuscript and the clusters (clusters are not included for estimating the overall signal) of each aldehyde.

Table S6 Main fragments of reported aldehydes measured by Vocus and used multiplication factor for aldehyde quantification.

Aldehydes	CAS	Parent ion	Fragment ion	Fraction (%) to the parent m/z	Multiplication factor used for comparison
Hexanal	66-25-1	$C_6H_{13}O^+$	$C_6H_{11}^+$	340%	4.4
Heptanal	111-71-7	$C_7H_{15}O^+$	$C_7H_{13}^+$	230%	3.3
Octanal	124-13-0	$C_8H_{17}O^+$	$C_8H_{15}^+$	96%	1.96
Nonanal	124-19-6	$C_9H_{20}O^+$	$C_9H_{17}^+$<br>$C_5H_9^+$	20%<br>(100%)*	2.2
Decanal	112-31-2	$C_{10}H_{21}O^+$	-	-	1

*not included for multiplication factor

The same setup was used to perform tests on aldehydes for the NO^+- CIMS. Table S7 shows the major fragments observed during the lab experiment. Due to low correlation with parent ions during the chamber studies, the signal from fragments were excluded from quantification, therefore, reported values in the chamber study are most likely higher.

Table S7 Major fragments of reported aldehydes measured by NO^+-CIMS

Aldehydes	CAS	Parent m/z	Fragment m/z	Fraction (%) to the parent m/z
Pentanal	110-62-3	85.065 ($C_5H_9O^+$)	57.071	65%
Hexanal	66-25-1	99.080 ($C_6H_{11}O^+$)	71.088	49%
Heptanal	111-71-7	113.096 ($C_7H_{13}O^+$)	85.101	21%
Octanal	124-13-0	127.111 ($C_8H_{15}O^+$)	109.101	12%
Nonanal	124-19-6	141.127 ($C_9H_{17}O^+$)	- *	-
Decanal	112-31-2	155.143 ($C_{10}H_{19}O^+$)	173.146	17%

*****For nonanal, fragments were not observed.

References

(1) Lopez-Hilfiker, F.; Zhu, L.; Cappellin, L. Calibrating Vocus PTR-TOF Sensitivity Using a Subset of VOC Standards.

(2) Salthammer, T.; Hohm, U.; Stahn, M.; Grimme, S. Proton-Transfer Rate Constants for the Determination of Organic Indoor Air Pollutants by Online Mass Spectrometry. *RSC Adv.* **2023**, *13* (26), 17856–17868. https://doi.org/10.1039/d3ra01705b.

(3) Titzmann, T.; Graus, M.; Müller, M.; Hansel, A.; Ostermann, A. Improved Peak Analysis of Signals Based on Counting Systems: Illustrated for Proton-Transfer-Reaction Time-of-Flight Mass Spectrometry. *Int. J. Mass Spectrom.* **2010**, *295* (1), 72–77. https://doi.org/10.1016/j.ijms.2010.07.009.

(4) Müller, M.; George, C.; D'Anna, B. Enhanced Spectral Analysis of C-TOF Aerosol Mass Spectrometer Data: Iterative Residual Analysis and Cumulative Peak Fitting. *Int. J. Mass Spectrom.* **2011**, *306* (1), 1–8. https://doi.org/10.1016/j.ijms.2011.04.007.

(5) Ernle, L.; Wang, N.; Bekö, G.; Morrison, G.; Wargocki, P.; Weschler, C. J.; Williams, J. Assessment of Aldehyde Contributions to PTR-MS *m* / *z* 69.07 in Indoor Air Measurements. *Environ. Sci. Atmospheres* **2023**, *3* (9), 1286–1295. https://doi.org/10.1039/D3EA00055A.
